# Epidemiological Insights into Colorectal Cancer Survival in Kazakhstan (2014–2023): A Retrospective Analysis Using the National Electronic Registry of Oncological Patients

**DOI:** 10.3390/cancers17142336

**Published:** 2025-07-14

**Authors:** Diyora Abdukhakimova, Altynay Beyembetova, Ayana Ablayeva, Ruslan Akhmedullin, Temirgali Aimyshev, Aigerim Biniyazova, Gulnur Zhakhina, Zhanar Orazbekova, Galiya Orazova, Abduzhappar Gaipov

**Affiliations:** 1 Department of Medicine, School of Medicine, Nazarbayev University, Astana 010000, Kazakhstan; dabdukhakimova@nu.edu.kz (D.A.); altynay.akhmedinova@nu.edu.kz (A.B.); ayana.ablayeva@nu.edu.kz (A.A.); ruslan.akhmedullin@nu.edu.kz (R.A.); temirgali.aimyshev@nu.edu.kz (T.A.); aigerim.biniyazova@nu.edu.kz (A.B.); gulnur.zhakhina@nu.edu.kz (G.Z.); 2 Department of Public Health and Hygiene, Astana Medical University, Astana 010000, Kazakhstan; zh.orazbekova@gmail.com (Z.O.); galiyaorazova@gmail.com (G.O.); 3 Clinical Academic Department of Internal Medicine, CF “University Medical Center”, Astana 010000, Kazakhstan

**Keywords:** colorectal cancer, epidemiology, incidence, mortality, survival, Cox regression

## Abstract

**Background**: Colorectal cancer continues to be a leading cause of cancer-related deaths worldwide; therefore, it is important to monitor its burden patterns. The aim of this study was to analyze the epidemiology and survival trends of colorectal cancer in Kazakhstan from 2014 to 2023. **Methods**: Data from Kazakhstan’s National Electronic Registry of Oncological Patients were analyzed using descriptive statistics. Overall survival at 3 and 5 years was estimated with Kaplan–Meier analysis. Multivariable Cox proportional hazards models were used to evaluate factors associated with survival, incorporating stratification by cancer stage and dividing follow-up into two-time intervals (first and subsequent years). Time-varying covariates were applied to address violations of the proportional hazards’ assumption. **Results**: The total sample size of the colorectal cancer cohort was 37,871 cases. The crude incidence rate remained stable until 2019, followed by a decline in 2020–2021, whereas all-cause mortality and prevalence had an overall increasing pattern. The highest crude all-cause mortality was in stage IV patients. Males had a higher crude mortality rate than females. However, females had slightly better overall survival than males. There was noticeably increased overall mortality among patients aged 75 and older. The time-varying Cox models showed that the effect of demographic and clinical variables, including comorbidity burden, varied over time. A higher Charlson Comorbidity Index was associated with reduced overall survival, specifically during the extended follow-up period. **Conclusions**: The findings highlight the critical need for targeted interventions, especially for older patients (≥75) with certain primary tumor locations, histological subtypes and comorbidities.

## Simple Summary

Early assessment of colorectal cancer’s temporal patterns in incidence, mortality and survival is important for accurate treatment and follow-up in the affected cohort. The aim of our retrospective study was to analyze cancer records of patients in Kazakhstan from 2014 to 2023. We found temporal trends in incidence, mortality, prevalence and survival. We identified that stage II was the most diagnosed, while stage IV colorectal cancer was the deadliest in the Kazakhstani population. Also, survival rates differed based on factors like age (≥75), gender, primary tumor location (rectum, right colon), presence of comorbidities and histological subtype. Still, further research is needed to better understand the prognostic and therapeutic implications of these covariates.

## 1. Introduction

Colorectal cancer (CRC) has attracted the attention of scientific communities as one of the leading causes of death in humans. CRC is the third most common cancer globally by incidence and shows high incidence rates among males [1,2]. It ranks second in cancer-related deaths among both men and women globally [1]. While incidence rates of colorectal cancer are almost three times higher in more developed countries than in those in transition, mortality rates vary less due to a higher average case fatality in lower Human Development Index (HDI) regions [2]. The generational changes in age-period cohort studies demonstrate that nutrition, obesity, and lifestyle factors are associated with an increasing pattern in incidence [2]. In contrast, effective cancer therapy and management approaches have increased survival rates in developed countries while decreasing mortality [2].

In Kazakhstan, CRC has emerged as one of the most diagnosed malignancies. According to Mauyenova et al., there was an upward trend in CRC incidence from 2009 to 2018, but there was no considerable difference in crude incidence rates between males (16.7 per 100,000) and females (17.2 per 100,000) [3]. However, researchers found that age-standardized incidence rates were evidently higher in males (21.9 per 100,000) than in females (15.9 per 100,000). This research also revealed geographic differences across different regions of Kazakhstan [3]. Contrary to incidence patterns, mortality rates from CRC in Kazakhstan follow the global trend of decline [4]. Still, authors note that Kazakhstan is a region with high mortality rates, with a standardized mortality rate of 10.2 per 100,000 population [4]. In addition, a study by Akhmedullin et al. showed increased CRC mortality in Kazakhstan from 2014 to 2022 with regional variations [5].

CRC screening has been conducted in Kazakhstan since the middle of 2011 with a target group of people aged 50–70 years every two years [3,6]. The CRC screening program involves a two-step process: firstly, patients undergo a fecal occult blood test, specifically the Hemoccult test, and those with positive results are advised by doctors to undergo a total colonoscopy [3,6]. These early detection procedures are conducted at specialized medical institutions such as city and district polyclinics [6]. Additionally, Zhylkaidarova et al. showed the important role CRC screening plays in monitoring prevalence trends [6]. Authors determined that introducing a nationwide screening program in 2011 led to early-stage detection and the upward trend in prevalence rates of colorectal cancer patients [6].

However, there are few studies that examine CRC survival in Kazakhstan, and there is limited understanding of how certain clinical and demographic factors influence the overall survival outcomes in the Kazakhstani CRC population.

Therefore, our study aimed to analyze the incidence, prevalence and all-cause mortality from 2014 to 2023 of CRC in Kazakhstan. In addition, we assessed overall survival by investigating the impact of key demographic and clinical factors on CRC patient outcomes.

## 2. Materials and Methods

### 2.1. Study Design and Selection Criteria

We conducted a retrospective cohort study using anonymized data on patients diagnosed with CRC from 2014 to 2023. The data were from the Electronic Registry of Oncological Patients (EROP) in Kazakhstan, where each patient is assigned a unique resident population number (RPN ID) for record linkage purposes [7]. Further details on the EROP database and methodology are available in our previous study [7]. EROP is the largest cancer repository in Kazakhstan, containing data from inpatient, outpatient, and dispensary medical records [7].

Missing data on age, sex, region, and place of residence were supplemented, where available, using records from the Unified National Electronic Health System (UNEHS) [8]. The dataset was provided in fully anonymized form, with no possibility of reidentification, including via the RPN ID. The Nazarbayev University Institutional Review Board determined that the study did not involve human participants, and informed consent was not required.

Duplicate records were removed using the RPN ID to ensure that each patient was counted only once. Supplementary Figure S1 presents the full patient selection procedure.

### 2.2. Exposure and Covariates

We obtained demographic and clinical data for CRC cases from the EROP and outpatient, inpatient, and death records available within the UNEHS. The dataset included the resident population number (RPN ID), age, sex, region, ethnicity, place of residence (urban or rural), social status, ICD-10 codes for primary diagnoses and comorbidities, ICD-O-3 histology and behavior codes, date of birth, and date of death. We categorized age into five groups: 18–44, 45–54, 55–64, 65–74, and ≥75 years.

We identified CRC cases consisting of both benign and malignant neoplasms by using ICD-10 codes (Supplementary Table S1). Comorbidity data were extracted from outpatient and inpatient records based on ICD-10 coding (Supplementary Table S2). We analyzed the burden of comorbidities using the Charlson Comorbidity Index (CCI), which uses weighted scores based on the mortality risk associated with specific conditions (Supplementary Table S2). We used the ICD-based algorithm developed by Ludwigson et al. [9], which was adapted based on the Quan et al. version of the CCI [10]. This methodology aligned with the aims of our study and ensured minimal code duplication across disease categories.

Overall, we had analyzed 18 conditions for CCI calculation: myocardial infarction, congestive heart failure, peripheral vascular disease, cerebrovascular disease, chronic obstructive pulmonary disease (COPD), other chronic pulmonary diseases, rheumatic disease, dementia, hemiplegia, tetraplegia, diabetes, diabetes with end-organ damage, moderate or severe kidney disease, mild liver disease, moderate or severe liver disease, peptic ulcer disease, and any malignancy, including leukemia and lymphoma. Since several studies have shown that inflammatory bowel disease (IBD) can alter the development and progression of colorectal cancer, we decided to include the overall analysis of the comorbidities [11]. The IBD was not used for CCI calculation.

Additionally, we excluded metastatic cancer and colorectal cancer diagnoses from comorbidities used for CCI calculation, as they may present extensions of the primary disease under investigation (CRC). Final CCI scores ranged from 0 to 10 and were categorized into four comorbidity levels for analytical purposes: no comorbidity (CCI = 0), low (CCI = 1–2), moderate (CCI = 3–4), and high (CCI ≥ 5).

Histological subtypes of colorectal neoplasms were classified using ICD-O-3 histology and behavior codes (Supplementary Table S3). These were grouped into the following categories: classical adenocarcinoma, mucinous adenocarcinoma, signet-ring cell carcinoma, squamous cell carcinoma not otherwise specified (NOS), neoplasms NOS, epithelial neoplasms NOS, and other specified types.

Primary tumor location was categorized based on ICD-10 codes, according to the classification systems presented in studies by Luhn et al., Majek et al., and Wang et al. (Supplementary Table S4) [12,13,14]. We classified primary tumor location into the following groups: left colon (descending colon), rectosigmoid junction, rectum, right colon (ascending colon), transverse colon, unspecified colon site and not applicable (benign, in situ, neoplasms of uncertain behavior). Due to inconsistencies in existing literature, the rectosigmoid junction was treated as a distinct category. Additionally, we decided to analyze the transverse colon separately because it embryologically spans both the right and left colon, which was consistent with the Majek et al. study [13].

We categorized ethnicity based on three major groups: Kazakh, Russian and Other (Uzbek, Ukrainian, etc.). Social status was classified as disabled, employed, housewife, other, pensioner and unemployed.

### 2.3. Outcome Assessment

We evaluated crude incidence, all-cause mortality, prevalence, and survival outcomes for patients diagnosed with CRC in Kazakhstan between 2014 and 2023. The date of the first diagnosed hospital case was considered the start of follow-up, with follow-up ending either at the date of death or at censoring on 31 December 2023. We calculated person-time at risk as the total sum of follow-up time contributed by all patients.

We calculated annual crude incidence and mortality rates by dividing the number of new CRC cases and all-cause deaths, respectively, by the average annual population for each calendar year. These incidence and mortality rates were calculated per 100,000 population. We obtained average annual population numbers for each year from the Bureau of National Statistics of the Agency for Strategic Planning and Reforms of the Republic of Kazakhstan [15].

We estimated crude point prevalence rates using a cumulative approach. For each year, the cumulative number of incident cases since 2014 was calculated, from which cumulative all-cause deaths were subtracted to approximate the number of individuals living with CRC. This figure was then divided by the average annual population for the respective year and multiplied by 100,000 to derive the point prevalence rate as of 31 December each year.

We also calculated age-standardized rates (ASR) for overall and sex-specific estimates of incidence (ASIR), mortality (ASMR) and prevalence (ASPR) using standard population from WHO [16].

### 2.4. Statistical Analysis

We performed data management and cleaning on a secure web server at Nazarbayev University using STATA version 16 MP2 (StataCorp, College Station, TX, USA). The study followed the Strengthening the Reporting of Observational Studies in Epidemiology (STROBE) guidelines. Demographic and clinical characteristics of the study population were summarized using descriptive statistics appropriate for the type and distribution of each variable (see Table 1, Table 2 and Table 3).

The distribution of age was assessed for normality using the skewness/kurtosis test. Both tests results were statistically significant (*p* < 0.0001); thus, age did not follow normal distribution. Therefore, we used the nonparametric Wilcoxon rank-sum test to compare the age distribution between patients who were alive and dead at the end of follow-up.

We conducted univariable and multivariable analysis to evaluate factors associated with overall survival (OS). Time to event was defined as the time in years from the date of diagnosis to the date of death or censoring at the last known follow-up. A *p*-value of 0.05 was used as a cut-off of significance throughout the analysis.

Survival distributions were estimated using the Kaplan–Meier method at 3-year and 5-year intervals. Group differences in survival were assessed using both the Log-rank test and the Wilcoxon–Gehan–Breslow test. We used both tests for a robust assessment and to account for potential non-proportionality in the survival curves.

We included in the multivariable Cox proportional hazards models those variables that were clinically meaningful and statistically significant in univariable analyses (*p* < 0.05, based on chi-squared tests). We also selected only one variable from each set of conceptually overlapping factors as conditions from the same comorbidity classification and CCI groups to exclude multicollinearity. Thus, although both mild and moderate/severe liver disease were individually associated with survival, only mild liver disease (*p* < 0.001) was included due to its stronger association and to reduce redundancy. Additionally, we calculated the variance inflation factors (VIFs) to assess multicollinearity, and all values were below 1.2, indicating no significant concern.

We tested the proportional hazards (PH) assumption using scaled Schoenfeld residuals and the Grambsch–Therneau global test. Due to evidence of non-proportionality, survival analyses were stratified by follow-up intervals: the first year after diagnosis and subsequent years. The PH assumption for cancer stage held during the first year but was violated in subsequent years. Therefore, all Cox regression models were stratified by stage, allowing the baseline hazard to vary across stage categories while estimating the effects of other covariates.

Still, further analysis showed that certain subgroups within categorical variables continued to violate the PH assumption. Therefore, we applied time-varying Cox models to accommodate time-dependent effects using the tvc () and texp () options in Stata, with log-transformed time specified for variables with PH violations. Stratification by stage was preserved in all time-varying models.

The final Cox models included demographic characteristics (e.g., age, sex, ethnicity), tumor features (e.g., primary tumor location, histology), and relevant comorbidities. To avoid multicollinearity, two separate models were specified: one incorporating individual comorbid conditions and another using composite CCI categories. Only one set was included per model (see Table 4 and Supplementary Table S5). Variables with missing or non-applicable values (e.g., “Missing” or “Not Indicated”) were excluded to ensure analytical clarity.

The total analytical sample comprised 32,439 patients. All individuals were included in the survival analysis for the first year of follow-up. For subsequent survival analysis beyond the first year, the sample included 20,046 individuals who remained at risk.

## 3. Results

### 3.1. Baseline Characterisitcs of the Study Cohort

The overall socio-demographic characteristics of the study cohort are summarized in Table 1, followed by medical characteristics (Table 2) and presence of comorbidities in Table 3. A total of 37,871 distinct colorectal cancer cases were included in the study sample. During the study period, there were 18,397 dead (48.58% of the total) and 19,474 alive patients (51.42% of the total).

The median age at first CRC diagnosis for those who were alive was 63.46 (IQR: 56.08–69.95), whereas the median age for those who were deceased was 66.95 (IQR: 59.20–75.28). The Wilcoxon rank-sum test demonstrated a statistically significant difference between the two groups (z = −32.28, *p* < 0.001), indicating that individuals in the deceased group experienced their first CRC diagnosis at an older age compared to those who were alive. Of this colorectal cancer cohort, 40.02% were Kazakh, 39.49% were Russian and 20.04% were Other (Uzbek, Ukrainian, etc.). Overall, 51.55% of the study cohort were female and 48.41% were male. The major histological subtype that comprised 68.64% of colorectal cancer patients was classical adenocarcinoma. The most common primary tumor location was the rectum (33.44%), followed by the left colon (28%) and right colon (14.77%).

Mortality rates per 1000 person-years (PY) were the highest among CRC patients aged 75 and above, accounting for 293.3 [95 CI: 285.3; 301.5], who comprised 20.18% of the total study cohort. The highest all-cause mortality rates per 1000 PY were detected in patients with the following characteristics: male, Russian, rural residence, pensioner, IV stage, signet-ring cell carcinoma, transverse colon as primary tumor location, and diabetes. The patients with CCI of 0 showed the highest mortality rate, 195.0 per 1000 PY [95% CI: 191.5; 198.5].

### 3.2. CRC Incidence, Prevalence and All-Cause Mortality Rate Among the Study Cohort

**Figure 1:**
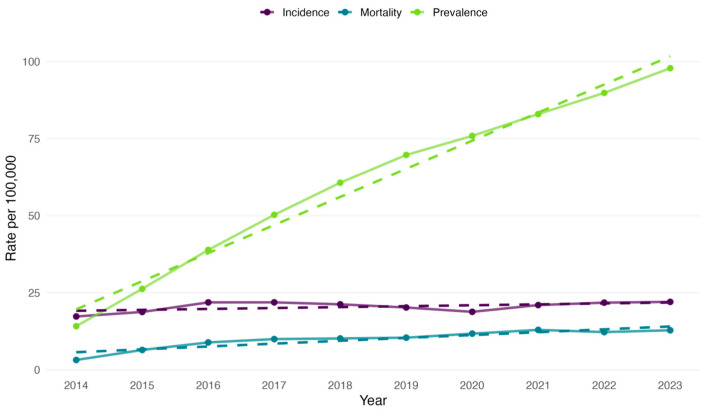
Incidence, prevalence and all-cause mortality rates of colorectal cancer in Kazakhstan per 100,000 population by year from 2014 to 2023.

We calculated the incidence rate of colorectal cancer in Kazakhstan per 100,000 population for each year from 2014 to 2023. The highest incidence rate was in 2023 (22.01 per 100,000), whereas the lowest was in 2014 (17.27 per 100,000) (Figure 1).

The all-cause mortality rate was the lowest in 2014 (3.16 per 100,000). Still, further, it increased through 2019 (10.39 per 100,000) and continued to rise in subsequent years. The highest mortality rate was observed in 2021 (12.91 per 100,000), with the second highest in 2023 (12.79 per 100,000) (Figure 1). Over the study period, the crude prevalence rate had an increasing pattern (Figure 1).

**Figure 2:**
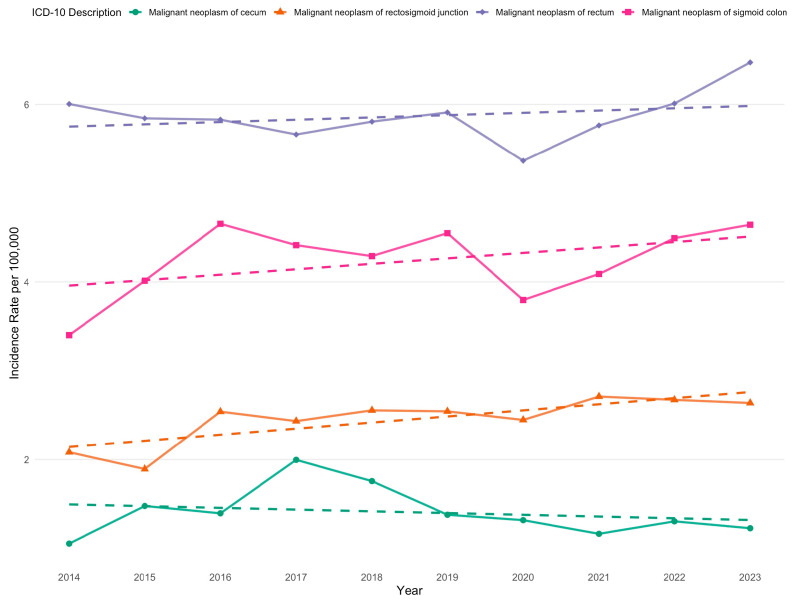
Crude incidence rates of the most common colorectal cancer subtypes per 100,000 population in Kazakhstan (2014–2023).

In addition, incidence rates were analyzed by specific ICD-10 codes corresponding to colorectal cancer subtypes. The highest incidence was observed for the following codes: C18.0 (malignant neoplasm of the cecum), C18.7 (malignant neoplasm of the sigmoid colon), C19 (malignant neoplasm of the rectosigmoid junction), and C20 (malignant neoplasm of the rectum) (Figure 2).

**Figure 3:**
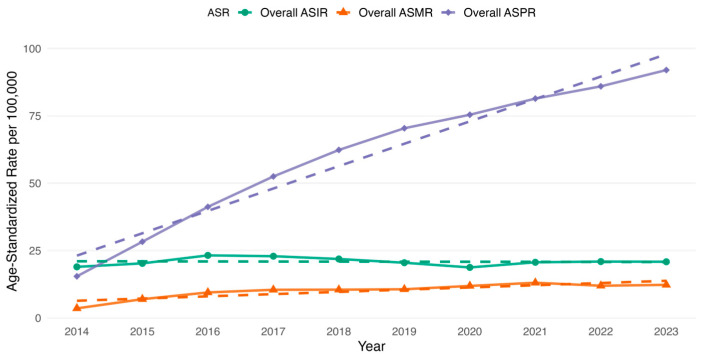
Age-standardized rates (ASR) of overall incidence (ASIR), prevalence (ASPR) and all-cause mortality rate (ASMR) of colorectal cancer in Kazakhstan per 100,000 population by year from 2014 to 2023.

**Figure 4:**
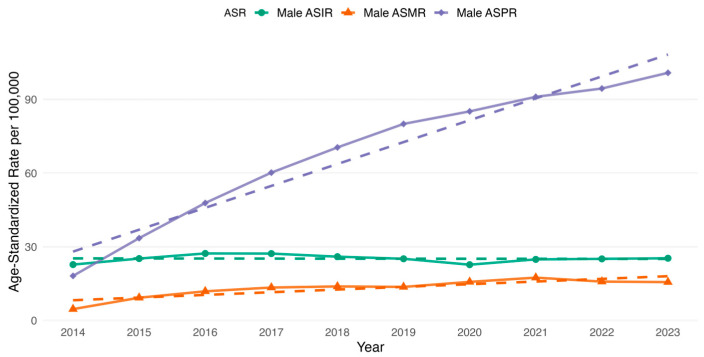
Age-standardized rates (ASR) of male-specific incidence (ASIR), prevalence (ASPR) and all-cause mortality rate (ASMR) of colorectal cancer in Kazakhstan per 100,000 population by year from 2014 to 2023.

**Figure 5:**
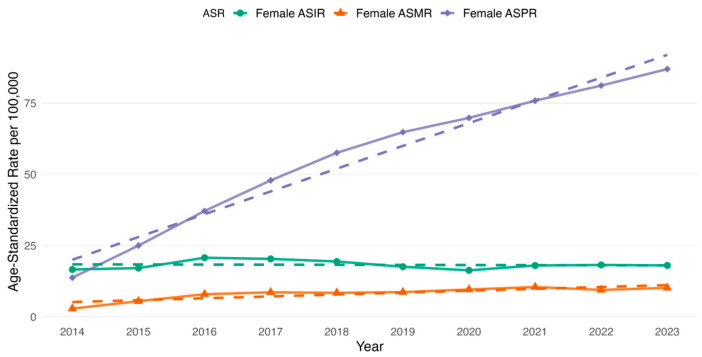
Age-standardized rates (ASR) of female-specific incidence (ASIR), prevalence (ASPR) and all-cause mortality rate (ASMR) of colorectal cancer in Kazakhstan per 100,000 population by year from 2014 to 2023.

We additionally examined the age-standardized rates for overall and sex-specific estimates of incidence, prevalence and mortality. The overall ASIR increased from 18.93 per 100,000 in 2014 to 20.81 per 100,000 in 2023 (Figure 3). The highest overall ASIR increase occurred in 2016 (23.19 per 100,000) and 2017 (22.88 per 100,000) (Figure 3). Following these years, there was a gradual decline in the overall ASIR from 2018 (21.85 per 100,000) that persisted until 2020 (18.71 per 100,000) inclusive (Figure 3). Further, the trend started to increase slowly (Figure 3). For the sex-specific ASIR, it was observed that male ASIR increased from 22.73 per 100,000 in 2014 to 25.31 per 100,000 in 2023, whereas for females it started at 16.60 per 100,000 in 2014 and reached 18.03 per 100,000 in 2023 (Figure 4 and Figure 5). The highest male-specific ASIR was 27.27 per 100,000 in 2016, whereas for female-specific ASIR it accounted for 20.73 per 100,000 in the same year. The lowest estimate for ASIR in both sexes was in 2020, having 22.69 per 100,000 for males and 16.31 per 100,000 for females.

The overall ASMR increased from 3.52 per 100,000 in 2014 to 12.25 per 100,000 in 2023 (Figure 3). The highest overall ASMR was 13.03 per 100,000 in 2021 (Figure 3). Similarly, the highest male-specific ASMR was 17.42 per 100,000 in 2021, whereas for female-specific ASMR, it was 10.44 per 100,000 in the same year (Figure 4 and Figure 5). The lowest estimate for ASMR in both sexes was in 2014, accounting for 4.60 per 100,000 for males and 2.88 per 100,000 for females (Figure 4 and Figure 5).

In addition to the observed changes in age-standardized rates, the ASPR demonstrated a consistent increase in the overall and sex-specific rates. For instance, the overall age-standardized prevalence increased from 15.41 per 100,000 in 2014 to 91.98 per 100,000 in 2023. Whereas, among males, the ASPR increased from 18.13 per 100,000 in 2014 to 100.81 per 100,000 in 2023. Similarly, among females, the ASPR rose from 13.72 per 100,000 to 86.94 per 100,000 over the same study period.

Furthermore, regional variation in colorectal cancer incidence and mortality was examined. Supplementary Figures S2 and S3 present the average colorectal cancer incidence and all-cause mortality rates across regions of Kazakhstan for the periods 2014–2017, 2018–2021, and 2022–2023. As detailed in Supplementary Table S6, regions such as Aktobe, Almaty, Atyrau, Jambyl, Kyzylorda, Mangystau, and Turkistan exhibited relatively stable CRC incidence rates throughout the study period. In contrast, regions including Akmola, Almaty City, Astana City, East Kazakhstan, Karaganda, Kostanay, North Kazakhstan, and Pavlodar demonstrated a consistent upward trend in incidence rates over time. By 2023, the highest incidence rates were observed in East Kazakhstan, Karaganda, Kostanay, and Pavlodar.

Similarly, all-cause mortality by regions had the same pattern (Table S7). Increasing mortality trends were recorded in Akmola, East Kazakhstan, Kostanay, North Kazakhstan, and Pavlodar. Whereas relatively stable mortality rates were in Almaty, Atyrau, Jambyl, Kyzylorda, Mangystau, and Turkistan. The highest all-cause mortality rate was observed in East Kazakhstan, followed by Kostanay, Karaganda, North Kazakhstan, and Pavlodar in 2023.

### 3.3. Survival Analysis

#### 3.3.1. Overall Survival Probability at 3 and 5 Years via Kaplan–Meier Analysis

**Figure 6:**
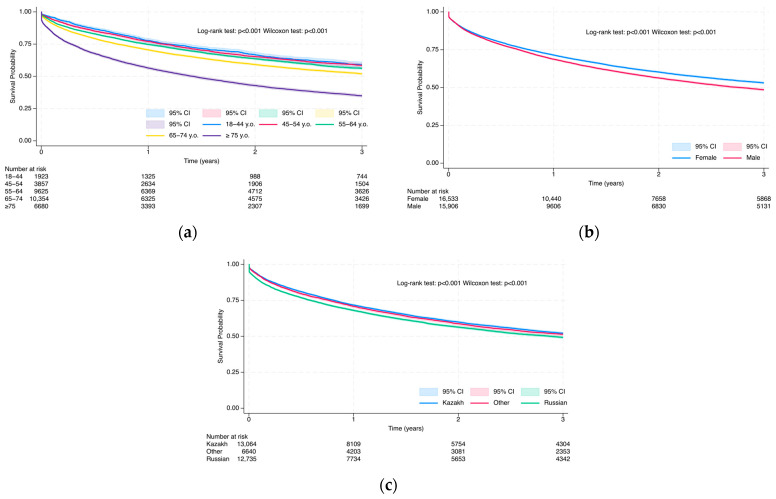
Kaplan–Meier survival curves due to all-cause mortality in colorectal cancer patients based on demographic characteristics: age group (**a**); sex (**b**); and ethnicity (**c**) during a 3-year follow-up.

**Figure 7:**
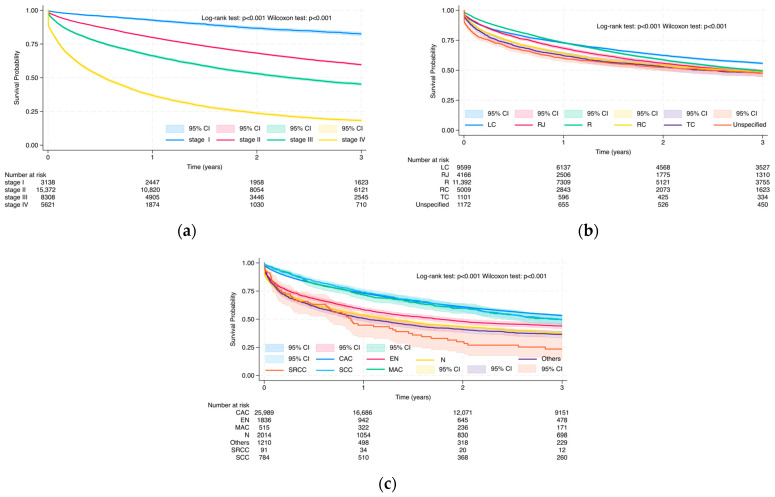
Kaplan–Meier survival curves due to all-cause mortality in colorectal cancer patients based on medical characteristics: stage (**a**); primary tumor location (**b**); and histological subtype (**c**) during 3-year follow-up.

**Figure 8:**
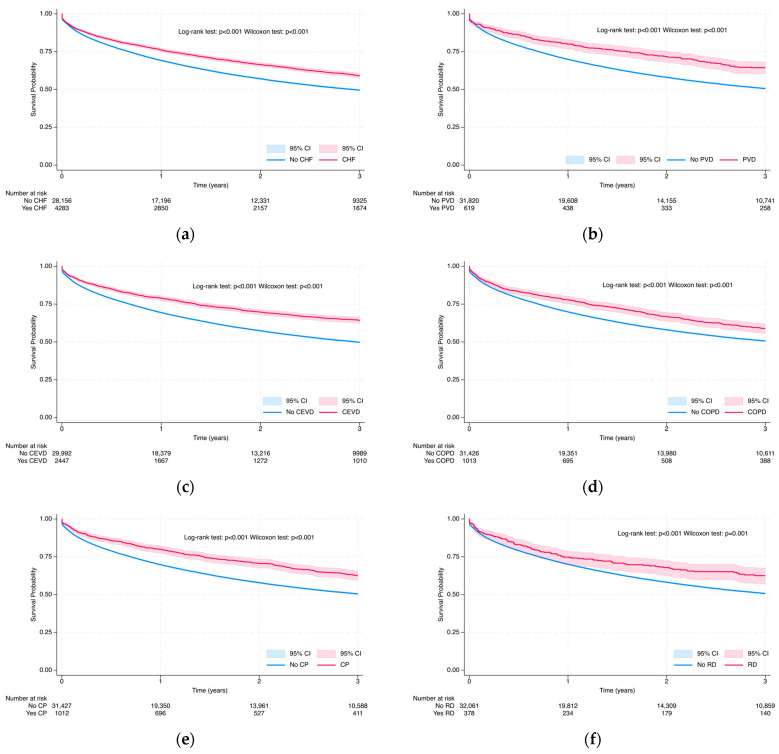

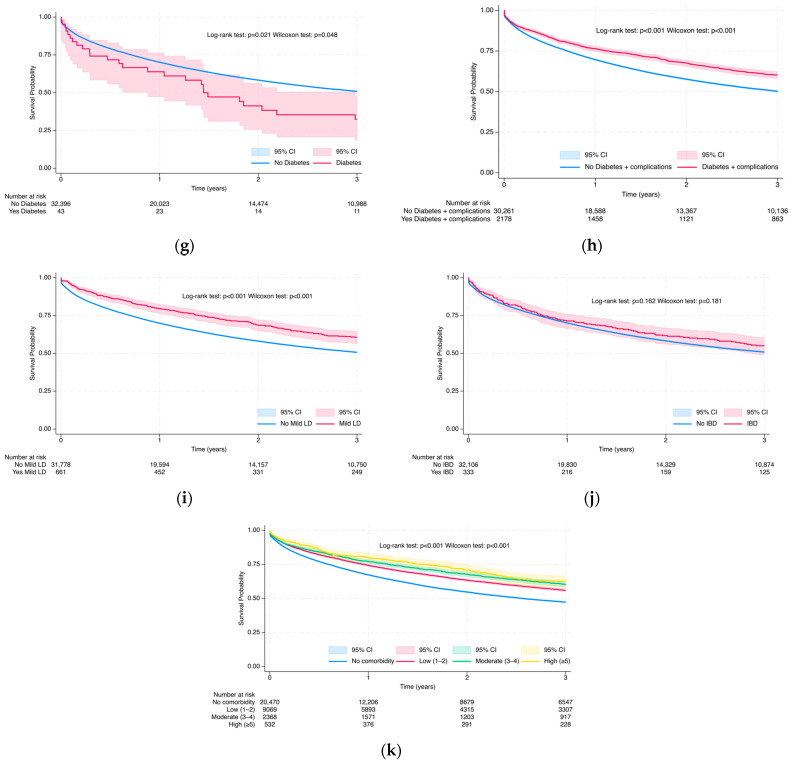
Kaplan–Meier survival curves due to all-cause mortality in colorectal cancer patients based on comorbidities and CCI group: congestive heart failure (**a**); peripheral vascular disease (**b**); cerebrovascular disease (**c**); chronic obstructive pulmonary disease (**d**); other chronic pulmonary disease (**e**); rheumatic disease (**f**); diabetes (**g**); diabetes with end organ damage (**h**); mild liver disease (**i**); inflammatory bowel disease (**j**); and CCI group (**k**) during 3-year follow-up.

Kaplan–Meier survival estimates at 3 years are presented in Figure 6, Figure 7 and Figure 8, whereas 5-year results are in Supplementary Figures S4–S6. Survival rates declined progressively over time, but still we observed differences in demographic and clinical subgroups. The Log-rank and Wilcoxon–Gehan–Breslow tests demonstrated significant differences in all-cause mortality among colorectal cancer patients across all demographic and medical characteristics (*p* < 0.001), as well as across all selected comorbidities and Charlson Comorbidity Index (CCI) groups, except inflammatory bowel disease (Log-rank test: *p* = 0.162; Wilcoxon–Gehan–Breslow test: *p* = 0.181) at the 3-year follow-up and at the 5-year follow-up (Log-rank test: *p* = 0.301; Wilcoxon–Gehan–Breslow test: *p* = 0.227) (Figure 6, Figure 7 and Figure 8 and S4–S6).

At 3 years, the survival rate among patients aged 18–44 was 59.1% (95% CI: 56.7–61.5%), decreasing progressively with age to 34.9% (95% CI: 33.7–36.1%) among those aged 75 and older (Figure 6a). A similar trend was observed at 5 years, with survival declining from 52.0% in the youngest group to 27.4% in the oldest (*p* < 0.001) (Figure S4A). Female patients demonstrated slightly better survival than males at both 3 years (53.1% vs. 48.5%) and 5 years (45.7% vs. 40.7%, *p* < 0.001) (Figure 6b and Figure S4B). Survival at 3 years was highest among patients of Russian ethnicity (43.4%), followed by Kazakh (43.0%) and other ethnicities (23.5%) (Figure 6c and Figure S4C). However, by 5 years, this trend shifted, with Kazakh patients demonstrating the highest survival rate (44.8%), followed by other ethnic groups (43.7%) and Russians (41.5%). These differences were statistically significant at both time points, as demonstrated by the Log-rank and Wilcoxon–Gehan tests (*p* < 0.001).

Stage at diagnosis was strongly associated with survival outcomes (Figure 7). Patients diagnosed with stage I colorectal cancer had the highest survival rates at both 3 years (82.5%) and 5 years (75.5%) (Figure 7a and Figure S5A). Survival declined progressively with advancing stage: stage II (59.7% at 3 years; 50.3% at 5 years), stage III (45.2%; 37.5%), and stage IV (18.4%; 15.6%) (*p* < 0.001 for both time points).

Primary tumor location significantly influenced prognosis. Patients with tumors in the left colon demonstrated better survival at 3 and 5 years (55.8% and 48.1%, respectively) compared to those with tumors in the rectum (49.3% and 40.7%), right colon (48.4% and 42.1%), rectosigmoid junction (48.3% and 41.2%), transverse colon (47.6% and 41.8%) and unspecified colon site (47.5% and 42.1%) (*p* < 0.001 at both time points) (Figure 7b and Figure S5B).

Survival by histological subtype was highest among patients with classical adenocarcinoma (3-year: 53.3%; 5-year: 45.1%), followed by squamous cell carcinoma, mucinous adenocarcinoma, epithelial neoplasms, other specified histologies, and unspecified neoplasms (*p* < 0.001 at both time points) (Figure 7c and Figure S5C). The lowest survival rates were observed in patients with signet ring cell carcinoma—23.5% at 3 years and 21.1% at 5 years.

Among heart-related comorbidities, CRC patients with peripheral vascular disease (PVD) exhibited the highest survival at both time points, with 64.4% at 3 years and 56.1% at 5 years, compared to 50.6% and 43.0% among those without PVD (Figure 8b and Figure S6B). Following this were patients with cerebrovascular disease (CEVD), showing survival rates of 64.2% and 54.1%, versus 49.8% and 41.6% for those without CEVD (Figure 8c and Figure S6C). Patients with congestive heart failure (CHF) had survival of 59.1% at 3 years and 50.4% at 5 years, whereas patients without CHF had 49.6% and 42.2%, respectively (Figure 8a and Figure S6A). Among chronic pulmonary diseases, CRC patients with other chronic pulmonary disease (CP) had higher survival rates at 3 years and 5 years, accounting for 62.6% and 54.3%, respectively, compared to 50.5% and 42.9% among those without CP (Figure 8e and Figure S6E). For chronic COPD, survival was 58.9% at 3 years and 51.3% at 5 years for patients with the condition, compared to 50.6% and 43.0% among those without COPD (Figure 8d and Figure S6D). At both the 3-year and 5-year survival marks, patients with rheumatoid disease showed higher survival (3-year: 62.6%; 5-year: 52.6%) compared to those without the condition (3-year: 50.7%; 5-year: 43.2%). A similar pattern was observed for diabetes with complications (3-year: 60.3%; 5-year: 51.0% vs. 50.2% and 42.7%) and mild liver disease (3-year: 60.6%; 5-year: 45.2% vs. 50.7% and 43.3%), where the presence of the condition was consistently associated with better survival (Figure 8h,i and Figure S6H,I). In all cases, survival was higher in patients with the condition present at both time points except diabetes without end-organ damage (Figure 8g and Figure S6G). For this condition, patients without diabetes had higher survival at 3 years (50.9%) and 5 years (43.3%) compared to those with the condition (32.4% and 22.2%, respectively). Overall, the presence of CP was associated with the highest 3-year survival among all comorbidities (62.6%), while PVD showed the highest 5-year survival (56.1%). In addition, survival increased in CCI groups with higher comorbidity scores at both 3 and 5 years. Patients in the high CCI group (≥5) had the highest survival (3-year: 62.6%; 5-year: 53.3%), while those with no comorbidities had the lowest survival (3-year: 47.3%; 5-year: 40.5%) (Figure 8k and Figure S6K).

#### 3.3.2. Multivariable Cox Proportional Hazards Regression Analysis

The results of the adjusted Cox regression models for CRC patients are in Table 4 and Table S5. Patients aged 75 years or older had a reduced risk of death during the first year (aHR = 0.86, 95% CI: 0.81–0.93), but a significantly increased risk in the following years (aHR = 2.10, 95% CI: 1.88–2.35). These estimates reflect a time-dependent association (Table 4). Compared to females, male patients exhibited a significantly higher risk of all-cause mortality during the first year of follow-up (adjusted hazard ratio [aHR], 1.15; 95% confidence interval [CI], 1.10–1.20; *p* < 0.001). However, this association was not significant in the subsequent follow-up period (aHR, 1.07; 95% CI, 0.98–1.17; *p* = 0.129). The results on Russian ethnicity compared to Kazakhs had an aHR with a 9% lower risk of death in the first year of follow-up (aHR = 0.91; 95% CI: 0.89–0.93; *p* < 0.001). However, in the subsequent follow-up years, the risk difference was not statistically significant (aHR = 1.03; 95% CI: 0.97–1.09; *p* = 0.284).

For primary tumor location, compared to the left colon, tumors located in the rectum were associated with a significantly increased risk of death during the first year of follow-up (aHR = 1.21; *p* < 0.001), but a significantly decreased risk in the subsequent years (aHR = 0.86; *p* = 0.006) (Table 4). Similarly, tumors located in the right colon (ascending colon) showed a modestly increased risk in the first year (aHR = 1.07; *p* < 0.001) and decreased risk after the first year (aHR = 0.84; *p* = 0.025).

Regarding histological subtypes, *epithelial neoplasms*, NOS were associated with a significantly decreased risk of death during the first year of follow-up (adjusted hazard ratio [aHR] 0.89, *p* < 0.001), but this association was not significant in subsequent years (Table 4). Similarly, there was an increased risk in estimates on *mucinous adenocarcinoma* during the first year (aHR 1.21, *p* = 0.002) with no significant association thereafter. Whereas, only neoplasms, NOS, demonstrated statistically significant associations with survival in both follow-up periods. For example, during the first year, the adjusted hazard ratio (aHR) was 0.86 (95% CI: 0.83–0.89, *p* < 0.001), while in subsequent years it was 0.80 (95% CI: 0.68–0.95, *p* = 0.012). These estimates for neoplasms, NOS, indicate a decreased risk of death compared to the reference group (classical adenocarcinoma) in the CRC cohort, a protective association that persisted despite evidence of proportional hazards violation, for which time-varying covariate modeling was applied.

Among comorbidities, three conditions—congestive heart failure, diabetes with complications, and mild liver disease—were significantly associated with overall survival in both the first year (*p* < 0.001, *p* = 0.015, *p* = 0.007) and subsequent years of follow-up (*p* < 0.001, *p* < 0.001, *p* = 0.009). These comorbidities showed aHRs indicating a protective effect during the first year but an increased risk during the later period. While, for instance, peripheral vascular disease and other pulmonary diseases demonstrated significant associations (*p* = 0.004, *p* = 0.002) with protective effects during the first year only, but no significant effects thereafter (*p* = 0.102, *p* = 0.077). Conversely, cerebrovascular disease and diabetes without end-organ damage were not significant in the first year but were significantly associated with increased risk during the subsequent years (aHR = 1.28, *p* = 0.005; aHR = 2.45, *p* < 0.001, respectively) (Table 4).

The Cox regression model that included demographic and clinical covariates along with CCI groups produced findings consistent with the model using individual comorbidities (Table S5). During the subsequent follow-up period (after the first year), increasing age was significantly associated with higher all-cause mortality risk, particularly among patients aged 55–64, 65–74, and 75 years or older. The estimates on male sex were significantly associated with increased mortality during the first year of follow-up (aHR = 1.14; *p* < 0.001), but not in subsequent periods. Russian ethnicity was associated with a protective effect during the first year (aHR < 1; *p* < 0.001), although the association was not statistically significant in the subsequent years. The results on primary tumor location showed that both the rectum and right colon were associated with higher mortality in the first year (aHRs > 1, *p* < 0.001, *p* < 0.001) but had a protective effect in the subsequent period (aHRs < 1, *p* = 0.014, *p* = 0.032), with statistically significant *p*-values in both intervals. In histological subtypes, only neoplasms, NOS, showed a consistent protective association with survival for both follow-up periods. Moreover, while higher CCI scores were associated with a lower risk of mortality during the first year, they were linked to increased mortality risk during the subsequent years. These estimates for CCI groups highlight the time-dependent impact of comorbidity burden on overall survival (Table S5).

## 4. Discussion

This study represents one of the most comprehensive investigations to date on the epidemiology of CRC in Kazakhstan, using UNEHS and EROP. Our findings indicate a rising trend in CRC incidence, prevalence, and all-cause mortality between 2014 and 2023 in Kazakhstan. In addition, our analysis indicated that, after accounting for differences in age structure, males consistently had higher ASIR, ASMR and ASPR than females. Therefore, we can observe that males are disproportionately affected by CRC, as reflected in higher age-standardized incidence, mortality, and prevalence rates.

Similarly, colorectal cancer incidence has an increasing trend globally [1]. It is believed that changes in lifestyle and important demographic and clinical risk factors contribute to such a pattern [1,2]. For instance, the latest analysis from the World Cancer Research Fund and the American Institute for Cancer Research shows that processed meat, alcoholic beverages, and body fatness increase colon cancer risk, whereas physical activity protects. These changes are the result of rising incomes, an older population and a rise in the total population residing in the country [1,2]. What is more important is that, in most countries, CRC is a leading cause of death for those under the age of 70 [2]. However, the majority of new CRC cases occur in low- and middle-income countries (LMICs), where the survival rate is considerably lower [2]. Therefore, this disease is known as a measure of socioeconomic growth, where incidence rates tend to increase with HDI levels in countries undergoing major development [2]. In the previous decade, Baltic countries, Russia, China, and Brazil have seen higher incidence and mortality [2,17]. In Canada, the UK, Denmark, and Singapore, incidence is rising while mortality falls [2]. In the US, Japan, and France, incidence and mortality are falling [2].

In addition, consistent with prior literature, several demographic and clinical characteristics analyzed in our study were substantially associated with overall survival among CRC patients. Our multivariable Cox regression estimates indicated time-dependent effects for critical factors such as age, gender, histological subtype, primary tumor location, and comorbidity status. These results add to current research that emphasizes the dynamic influence of these covariates on CRC survival [18,19,20,21,22,23,24,25,26,27,28,29,30,31,32,33,34,35,36,37,38].

The age-related increase in mortality risk during longer follow-up aligns with previous studies showing that older age is a consistent predictor of poorer outcomes in CRC patients. These results may persist in different studies, potentially due to reduced physiological reserve and higher prevalence of chronic conditions in older-aged patients. The sex-related differences observed, such as higher early crude all-cause mortality risk among male patients, may be linked to delayed care-seeking or biological differences between the two sexes. Similarly, the time-varying associations of tumor location (rectum and right colon) and histology (neoplasms, NOS) support previous research indicating that anatomical site and tumor biology influence disease progression and treatment response.

Our results in analyzing the association between the overall survival of the CRC cohort with certain comorbidities were consistent with prior studies. For example, Morishima et al. found that comorbid conditions evidently increased all-cause mortality, even after adjusting for age, sex, and stage [24]. Michalopoulou et al. similarly reported elevated excess death rates among CRC patients with non-cancer comorbidities compared to those without [25]. Diabetes has been associated with delayed diagnosis, altered symptom presentation, and increased cancer recurrence risk [25]. Rubio et al. identified cerebrovascular disease, COPD, and diabetes as independent risk factors for decreased survival in CRC patients, with effects differing by cancer stage [26]. The presence of comorbidities was associated with reduced survival in our study cohort. Notably, CHF, diabetes with complications, and mild liver disease exhibited a protective effect in the first year, followed by increased mortality risk during longer-term follow-up. Our analysis of the CCI groups further supports this pattern. During the first year, higher CCI scores showed a decreasing protective effect; however, during subsequent years, mortality risk rose proportionally with the CCI group (aHRs = 1.48, 1.78, and 2.04 for increasing CCI categories, all *p* < 0.001; Table S5). The observed shift in the impact of CCI strengthens findings from studies that found that comorbidities may initially prompt closer clinical monitoring but eventually contribute to long-term mortality. Overall, these results emphasize the importance of accounting for time-dependent effects when assessing survival predictors in CRC populations.

Additionally, Tamraz et al. pointed out that imaging studies and colonoscopies are performed on people with multiple comorbidities when they receive an abnormal test result or certain signs, which could help doctors make a CRC diagnosis earlier [27]. Suenghataiphorn et al. found that dementia lowers the risk of inpatient death and other clinical outcomes in colorectal cancer patients [28]. These results can be explained by several factors, including the possibility that dementia patients with colorectal cancer will receive less aggressive treatment; neurodegenerative diseases may decrease acetylcholine production [39], which may lessen its impact on rapidly proliferating cancer cells; and dementia patients’ silent amyloid-forming protein generation may trigger an innate immune response and accelerate the death of cancerous cells [28,40]. Also, CRC patients with dementia are less likely to have hypertension, hyperlipidemia, diabetes, obesity, and chronic renal disease [26]. Lower detection and neglect, not lower risk of certain co-morbidities, may explain this result [26]. However, in our study, dementia was not a statistically significant factor-based chi-squared test, so we cannot draw conclusions on its risk on overall survival like was performed by Suenghataiphorn et al. Therefore, it could be concluded that certain comorbidities may influence the timeliness and appropriateness of therapeutic interventions, which in turn influences overall survival of the CRC cohort.

Additionally, our study also revealed observable regional disparities in both CRC incidence and all-cause mortality rates across Kazakhstan. Regions such as East Kazakhstan, Karaganda, Kostanay, and Pavlodar consistently exhibited the highest incidence and mortality rates by the end of the study period. Conversely, regions including Almaty, Atyrau, Jambyl, Kyzylorda, Mangystau, and Turkistan showed relatively stable trends over time. Schmocker et al. intended to determine whether the distance from a treatment center affects colorectal cancer stage at diagnosis and mortality [37]. They discovered that increasing distance from the treating facility resulted in a markedly higher clinical TNM stage, longer time to surgery, and higher mortality [37]. These findings imply that rural patients face large differences in access to cancer care, which may indicate wider regional disparities in access to healthcare and resource allocations [37]. The regional disparities in CRC burden in Kazakhstan indicate the need for targeted interventions in the northern and eastern regions. Our findings suggest an unequal geographic distribution of CRC burden, potentially reflecting differences in healthcare access as noted by Schmocker et al. [37].

### Limitations

There are several limitations to this study. Key demographic and clinical variables, including sex, ethnicity, social status and region, were missing for some CRC patients analyzed. This limited our ability to fully assess their impact on survival outcomes for the whole cohort of the study. However, this missing information had minimal influence on the estimation of incidence, prevalence and all-cause mortality trends.

Missing data were not incorporated into the Kaplan–Meier and Cox analyses, although they were retained in the dataset for determining crude mortality rates to preserve the full cohort size. Shi et al. suggests that missingness in clinical datasets is often driven by healthcare delivery practices rather than occurring at random [41,42,43,44,45,46]. Authors point out that while methods such as multiple imputation [47], complete case analysis, and inverse probability weighting (IPW) [48] are commonly used, they carry assumptions that can introduce bias [41]. Since missing values were not explicitly addressed in this study, there is potential for bias if their presence is systematically related to clinical characteristics or access to care. Future research should incorporate methods that model the missing data process to improve the validity and interpretability of findings.

Despite these limitations, our study provides important insights into CRC incidence, prevalence and all-cause mortality trends in Kazakhstan, using large-scale administrative health data. We recommend that the government of Kazakhstan enhance national surveillance systems and targeted interventions. Additionally, we suggest incorporating routine genetic testing, such as for Lynch Syndrome in newly diagnosed CRC patients, into clinical practice. These interventions would improve the identification of inherited cancer risk and familial risk assessment and inform personalized treatment strategies. Moreover, enhancing the management of colorectal cancer and comorbid conditions such as CHF, diabetes with complications and mild liver disease will be critical to improving patient survival outcomes.

## 5. Conclusions

This study investigates CRC epidemiology in Kazakhstan using large-scale administrative data from 2014 to 2023. Comprehensive analysis was performed to find the association between a wide range of factors and overall survival in the CRC Kazakhstani cohort. We observed an increasing trend in incidence, prevalence, and all-cause mortality during the whole study period. We determined that older age, certain primary tumor locations and histological subtypes are substantially associated with overall survival, with time-varying effects observed in Cox models stratified by cancer stage to account for its influence. For example, the higher CCI group had reduced survival probabilities, particularly during long-term follow-up. Thus, our results underscore the need for enhanced comorbidity assessment, targeted interventions and early CRC diagnosis to improve patient outcomes.

## Figures and Tables

**Table 1:** Socio-demographic characteristics and all-cause mortality rates of patients with colorectal cancer between 2014 and 2023.

	Total *n* = 37,871	Alive *n* = 19,474 (51.42)	Dead *n* = 18,397 (48.58)	*p*-Value	Mortality Rate per 1000 PY [95%CI]
Age, median (IQR)	65.03 (57.54–72.50)	63.46 (56.08–69.95)	66.95 (59.20–75.28)	<0.001	-
Age groups, *n* (%)				<0.001	
18–44	2350 (6.21)	1430 (7.34)	920 (5.00)		130.1 [122.0; 138.8]
45–54	4512 (11.91)	2687 (13.80)	1825 (9.92)		131.7 [125.8; 137.9]
55–64	11,321 (29.89)	6434 (33.04)	4887 (26.56)		142.8 [138.8; 146.8]
65–74	12,045 (31.81)	6284 (32.27)	5761 (31.31)		178.8 [174.3; 183.5]
≥75	7643 (20.18)	2639 (13.55)	5004 (27.20)		293.3 [285.3; 301.5]
Sex, *n* (%)				<0.001	
Female	19,524 (51.55)	10,563 (54.24)	8961 (48.71)		159.2 [155.9; 162.5]
Male	18,335 (48.41)	8905 (45.73)	9430 (51.26)		196.1 [192.2; 200.1]
Not indicated	12 (0.03)	6 (0.03)	6 (0.03)		116.1 [52.1; 258.4]
Ethnicity, *n* (%)				<0.001	
Kazakh	15,156 (40.02)	8173 (41.97)	6983 (37.96)		171.8 [167.8; 175.9]
Russian	14,954 (39.49)	7376 (37.88)	7578 (41.19)		182.3 [178.2; 186.4]
Other	7589 (20.04)	3856 (19.80)	3733 (20.29)		171.9 [166.5; 177.6]
Missing	172 (0.45)	69 (0.35)	103 (0.56)		203.7 [167.9; 247.1]
Residence, *n* (%)				<0.001	
Rural	8545 (22.56)	4158 (21.35)	4387 (23.85)		198.2 [192.4; 204.2]
Urban	23,710 (62.61)	12,480 (64.09)	11,230 (61.04)		169.1 [166.0; 172.2]
Missing	5616 (14.83)	2836 (14.56)	2780 (15.11)		175.1 [168.7; 181.8]
Social Status, *n* (%)				<0.001	
Disabled	496 (1.31)	239 (1.23)	257 (1.40)		185.8 [164.4; 209.9]
Employed	5148 (13.59)	2974 (15.27)	2174 (11.82)		134.3 [128.8; 140.1]
Housewife	887 (2.34)	512 (2.63)	375 (2.04)		149.5 [135.1; 165.5]
Other	3643 (9.62)	1926 (9.89)	1717 (9.33)		172.9 [164.9; 181.2]
Pensioner	19,327 (51.03)	8777 (45.07)	10,550 (57.35)		216.0 [211.9; 220.1]
Unemployed	2957 (7.81)	1631 (8.38)	1326 (7.21)		166.1 [157.4; 175.3]
Missing	5413 (14.29)	3415 (17.54)	1998 (10.86)		113.6 [108.7; 118.7]

**Table 2:** Medical characteristics and all-cause mortality rates of patients with colorectal cancer between 2014 and 2023.

	Total *n* = 37,871	Alive *n* = 19,474 (51.42)	Dead *n* = 18,397 (48.58)	*p*-Value	Mortality Rate per 1000 PY [95%CI]
Stages, *n* (%)				<0.001	
I	3402 (8.98)	2669 (13.71)	733 (3.98)		57.9 [53.9; 62.3]
II	16,419 (43.36)	9289 (47.70)	7130 (38.76)		142.3 [139.0; 145.6]
III	9025 (23.83)	3945 (20.26)	5080 (27.61)		221.9 [215.9; 228.1]
IV	5893 (15.56)	1278 (6.56)	4615 (25.09)		569.5 [553.3; 586.2]
Not applicable	268 (0.71)	196 (1.01)	72 (0.39)		73.9 [58.6; 93.1]
Missing	2864 (7.56)	2097 (10.77)	767 (4.17)		79.2 [73.8; 85.0]
Histological subtypes, *n* (%)				<0.001	
Classical adenocarcinoma	25,996 (68.64)	13,447 (69.05)	12,549 (68.21)		176.2 [173.1; 179.3]
Epithelial neoplasms, NOS	1840 (4.86)	824 (4.23)	1016 (5.52)		256.9 [241.5; 273.2]
Mucinous adenocarcinoma	515 (1.36)	250 (1.28)	265 (1.44)		191.6 [169.9; 216.1]
Neoplasms, NOS	2022 (5.34)	625 (3.21)	1397 (7.59)		230.7 [218.9; 243.1]
Other specified types	1210 (3.20)	489 (2.51)	721 (3.92)		359.3 [334.0; 386.5]
Signet-ring cell carcinoma	91 (0.24)	28 (0.14)	63 (0.34)		444.8 [347.5; 569.4]
Squamous cell carcinoma	784 (2.07)	396 (2.03)	388 (2.11)		187.1 [169.3; 206.6]
Missing	5413 (14.29)	3415 (17.54)	1998 (10.86)		113.6 [108.7; 118.7]
Primary tumor location, *n* (%)				<0.001	
Left colon (descending colon)	10,603 (28.00)	5707 (29.31)	4896 (26.61)		159.5 [155.1; 164.0]
Rectosigmoid junction	4545 (12.00)	2207 (11.33)	2338 (12.71)		197.2 [189.4; 205.4]
Rectum	12,664 (33.44)	6194 (31.81)	6470 (35.17)		185.4 [180.9; 189.9]
Right colon (ascending colon)	5594 (14.77)	2695 (13.84)	2899 (15.76)		201.6 [194.3; 209.0]
Transverse colon	1218 (3.22)	588 (3.02)	630 (3.42)		209.8 [194.0; 226.8]
Unspecified colon site	1408 (3.72)	649 (3.33)	759 (4.13)		162.8 [151.6; 174.8]
Not applicable (benign, in situ, uncertain behavior neoplasms)	1839 (4.86)	1434 (7.36)	405 (2.20)		82.2 [74.5; 90.6]

**Table 3:** Comorbidities and all-cause mortality rates of patients with colorectal cancer between 2014 and 2023.

	Total *n* = 37,871	Alive *n* = 19,474 (51.42)	Dead *n* = 18,397 (48.58)	*p*-Value	Mortality Rate per 1000 PY [95%CI]
Comorbidities					
Myocardial infarction, *n* (%)	777 (2.05)	408 (2.10)	369 (2.01)	0.540	161.5 [145.8; 178.8]
Congestive heart failure, *n* (%)	5046 (13.32)	2884 (14.81)	2162 (11.75)	<0.001	138.4 [132.7; 144.4]
Peripheral vascular disease, *n* (%)	764 (2.02)	479 (2.46)	285 (1.55)	<0.001	111.0 [98.8; 124.7]
Cerebrovascular disease, *n* (%)	3003 (7.93)	1910 (9.81)	1093 (5.94)	<0.001	111.6 [105.2; 118.4]
Chronic obstructive pulmonary disease (COPD), *n* (%)	1228 (3.24)	727 (3.73)	501 (2.72)	<0.001	130.9 [120.0; 142.9]
Other chronic pulmonary diseases, *n* (%)	1210 (3.20)	747 (3.84)	463 (2.52)	<0.001	120.7 [110.2; 132.2]
Rheumatic disease, *n* (%)	464 (1.23)	298 (1.53)	166 (0.90)	<0.001	119.8 [102.9; 139.5]
Dementia, *n* (%)	95 (0.25)	46 (0.24)	49 (0.27)	0.558	130.8 [98.9; 173.1]
Hemiplegia, tetraplegia, *n* (%)	140 (0.37)	69 (0.35)	71 (0.39)	0.612	198.5 [157.3; 250.5]
Diabetes, *n* (%)	49 (0.13)	16 (0.08)	33 (0.18)	0.009	313.3 [222.7; 440.7]
Diabetes with end-organ damage, *n* (%)	2554 (6.74)	1472 (7.56)	1082 (5.88)	<0.001	137.1 [129.1; 145.5]
Moderate or severe kidney disease, *n* (%)	3054 (8.06)	1577 (8.10)	1477 (8.03)	0.804	160.5 [152.5; 168.9]
Mild liver disease, *n* (%)	829 (2.19)	499 (2.56)	330 (1.79)	<0.001	135.6 [121.7; 151.0]
Moderate or severe liver disease, *n* (%)	60 (0.16)	21 (0.11)	39 (0.21)	0.011	240.2 [175.5; 328.8]
(Peptic) Ulcer disease, *n* (%)	1148 (3.03)	596 (3.06)	552 (3.00)	0.734	171.6 [157.8; 186.5]
Any malignancy, including leukemia and lymphoma *, *n* (%)	2547 (6.73)	1350 (6.93)	1197 (6.51)	0.098	187.6 [177.3; 198.5]
HIV/AIDS, *n* (%)	4 (0.01)	3 (0.01)	1 (0.01)	0.345	67.2 [9.5; 477.1]
Inflammatory bowel disease, *n* (%)	429 (1.13)	242 (1.24)	187 (1.02)	0.038	143.0 [123.9; 165.0]
CCI group				<0.001	
No comorbidity (0)	22,816 (60.25)	11,101 (57.00)	11,715 (63.68)		195.0 [191.5; 198.5]
Low (1–2)	11,132 (29.39)	6101 (31.33)	5031 (27.35)		156.2 [152.0; 160.6]
Moderate (3–4)	3163 (8.35)	1835 (9.42)	1328 (7.22)		137.4 [130.3; 145.0]
High (≥5)	760 (2.01)	437 (2.24)	323 (1.76)		130.0 [116.5; 145.0]

Note: * Does not include non-melanoma skin cancer, colorectal cancer.

**Table 4:** Association between demographic and medical parameters and all-cause mortality rates in colorectal cancer patients (2014–2023).

	First Year of Follow-Up		Subsequent Years	
	Hazard Ratio (95% CI)	*p*-Value	Hazard Ratio (95% CI)	*p*-Value
Age group				
18–44	reference		reference	
45–54	0.92 (0.85–0.99) ^†^	**0.033** ^†^	1.04 (0.92–1.17)	0.521
55–64	0.89 (0.83–0.95) ^†^	**0.001** ^†^	1.15 (1.03–1.29)	**0.010**
65–74	0.88 (0.83–0.94) ^†^	**0.001** ^†^	1.38 (1.23–1.54)	**<0.001**
≥75	0.86 (0.81–0.93) ^†^	**<0.001** ^†^	2.10 (1.88–2.35)	**<0.001**
Sex				
Female	reference		reference	
Male	1.15 (1.10–1.20)	**<0.001**	1.07 (0.98–1.17) ^†^	0.129 ^†^
Ethnicity				
Kazakh	reference		reference	
Other	1.00 (0.90–1.01) ^§^	0.928 ^§^	0.98 (0.92–1.04)	0.534
Russian	0.91 (0.89–0.93) ^†^	**<0.001** ^†^	1.03 (0.97–1.09)	0.284
Primary tumor location				
Left colon (descending colon)	reference		reference	
Rectosigmoid junction	1.09 (1.05–1.13) ^†^	**<0.001** ^†^	0.86 (0.75–1.00) ^§^	0.053 ^§^
Rectum	1.21 (1.17–1.25) ^†^	**<0.001** ^†^	0.86 (0.80–0.96) ^†^	**0.006** ^†^
Right colon (ascending colon)	1.07 (1.04–1.11) ^†^	**<0.001** ^†^	0.84 (0.72–0.98) ^§^	**0.025** ^§^
Transverse colon	1.03 (0.98–1.09) ^§^	0.252 ^§^	0.85 (0.63–1.14) ^§^	0.273 ^§^
Unspecified colon site	0.94 (0.90–0.99) ^§^	**0.020** ^§^	1.09 (0.85–1.40) ^§^	0.511 ^§^
Histological subtypes				
Classical adenocarcinoma	reference		reference	
Epithelial neoplasms, NOS	0.89 (0.85–0.92) ^†^	**<0.001** ^†^	0.84 (0.66–1.06) ^§^	0.153 ^§^
Mucinous adenocarcinoma	1.21 (1.07–1.37) ^†^	**0.002** ^†^	0.99 (0.72–1.38) ^§^	0.980 ^§^
Neoplasms, NOS	0.86 (0.83–0.89) ^†^	**<0.001** ^†^	0.80 (0.68–0.95) ^†^	**0.012** ^†^
Other specified types	0.96 (0.92–1.01) ^†^	0.095 ^†^	0.76 (0.56–1.05) ^§^	0.101 ^§^
Signet-ring cell carcinoma	1.03 (0.88–1.22) ^§^	0.068 ^§^	0.48 (0.16–1.45) ^§^	0.196 ^§^
Squamous cell carcinoma, NOS	1.18 (1.02–1.36) ^§^	0.101 ^§^	0.91 (0.69–1.20) ^§^	0.521 ^§^
Comorbidities				
Absent comorbidity	reference		reference	
Congestive heart failure	0.82 (0.76–0.87)	**<0.001**	1.39 (1.22–1.58) ^†^	**<0.001** ^†^
Peripheral vascular disease	0.76 (0.64–0.92)	**0.004**	0.86 (0.72–1.03)	0.102
Cerebrovascular disease	0.96 (0.92–1.01) ^†^	0.171 ^†^	1.28 (1.08–1.52) ^†^	**0.005** ^†^
Chronic obstructive pulmonary disease (COPD)	0.95 (0.88–1.02) ^†^	0.193 ^†^	0.87 (0.76–1.00)	0.058
Other chronic pulmonary diseases	0.79 (0.68–0.92)	**0.002**	0.88 (0.76–1.01)	0.077
Rheumatic disease	1.06 (0.86–1.31)	0.555	0.86 (0.67–1.11)	0.254
Diabetes	1.59 (0.96–2.65)	0.071	2.45 (1.48–4.07)	**0.001**
Diabetes with end-organ damage	0.89 (0.81–0.98)	**0.015**	1.37 (1.15–1.63)^†^	**<0.001** ^†^
Mild liver disease	0.79 (0.66–0.94)	**0.009**	1.51 (1.12–2.03) ^†^	**0.007** ^†^
Inflammatory bowel disease	1.16 (0.94–1.42)	0.154	1.05 (0.84–1.33)	0.659

Note: Adjusted hazard ratios (aHRs) and 95% confidence intervals (CIs) are derived from multivariable Cox regression models, adjusted for all covariates listed. Models were stratified by stage due to proportional hazards (PH) violation. Time-varying Cox (TVC) models were used where applicable. Results are presented separately for the first year of follow-up and the subsequent years to account for time-dependent effects. ^†^ PH assumption violated; TVC estimate reported. ^§^ PH assumption not violated, but TVC applied for consistency across variable categories. **Bold *p*-values** indicate statistical significance (*p* < 0.05).

## Data Availability

All data related to this study are available from Republican Center for Electronic Health of the Ministry of Health of the Republic of Kazakhstan, but restrictions apply to the availability of these data, which were used under the contract-agreement for the current study, and so are not publicly available. Data are, however, available from the corresponding author (abduzhappar.gaipov@nu.edu.kz) upon reasonable request and with permission of Ministry of Health of the Republic of Kazakhstan.

## Supplementary Materials

The following supporting information can be downloaded at https://www.mdpi.com/article/10.3390/cancers17142336/s1, Table S1: The ICD-10 codes used to identify colorectal cancer patients; Table S2: ICD-10 codes used to define comorbidities; Table S3: The number of colorectal cancer patients with different histological subtypes; Table S4: ICD-10 codes used to define primary tumor location; Table S5: Association between demographic and medical parameters and all-cause mortality rates in colorectal cancer patients (2014–2023); Table S6: Incidence rate of colorectal cancer in the regions of Kazakhstan by years in 2014–2023; Table S7: All-cause mortality rate of the colorectal cancer cohort in the regions of Kazakhstan by years in 2014–2023; Figure S1: Colorectal cancer cohort selection diagram; Figure S2: Average incidence rate of colorectal cancer in the regions of Kazakhstan per 100,000 population: (a) 2014–2017, (b) 2018–2021, (c) 2022–2023; Figure S3: Average all-cause mortality rate of colorectal cancer in the regions of Kazakhstan per 100,000 population: (a) 2014–2017, (b) 2018–2021, (c) 2022–2023; Figure S4: Kaplan–Meier survival curves due to all-cause mortality in colorectal cancer patients based on demographic characteristics: age group (a); sex (b); and ethnicity (c) during a 5-year follow-up; Figure S5: Kaplan–Meier survival curves due to all-cause mortality in colorectal cancer patients based on medical characteristics: stage (a); primary tumor location (b); and histological subtype (c) during 5-year follow-up; Figure S6: Kaplan–Meier survival curves due to all-cause mortality in colorectal cancer patients based on comorbidities and CCI group: congestive heart failure (a); peripheral vascular disease (b); cerebrovascular disease (c); chronic obstructive pulmonary disease (d); other chronic pulmonary disease (e); rheumatic disease (f); diabetes (g); diabetes with end organ damage (h); mild liver disease (i); inflammatory bowel disease (j); and CCI group (k) during 5-year follow-up.

## Abbreviations

The following abbreviations are used in this manuscript:

aHR	Adjusted Hazard Ratio
ASIR	Age-Standardized Incidence Rate
ASMR	Age-Standardized Mortality Rate
ASPR	Age-Standardized Prevalence Rate
ASR	Age-Standardized Rate
CAC	Classical Adenocarcinoma
CCI	Charlson Comorbidity Index
CEVD	Cerebrovascular Disease
CHF	Congestive Heart Failure
CI	Confidence Interval
COPD	Chronic Obstructive Pulmonary Disease
CP	Other Chronic Pulmonary Diseases
CRC	Colorectal Cancer
EN	Epithelial Neoplasms
EROP	Electronic Registry of Oncological Patients
HDI	Human Development Index
IBD	Inflammatory Bowel Disease
ICD-10	International Classification of Diseases, Tenth Revision
ICD-O-3	International Classification of Disease for Oncology by 3rd Edition
IQR	Interquartile Range
IPW	Inverse Probability Weighting
LC	Left Colon
LD	Liver Disease
MAC	Mucinous Adenocarcinoma
NOS	Not Otherwise Specified
OS	Overall Survival
PH	Proportional Hazards
PY	Person-Years
PVD	Peripheral Vascular Disease
R	Rectum
RC	Right Colon
RD	Rheumatic Disease
RJ	Rectosigmoid Junction
RPN ID	Resident Population Number
SCC	Squamous Cell Carcinoma
SRCC	Signet Ring Cell Carcinoma
STROBE	Strengthening the Reporting of Observational Studies in Epidemiology
TC	Transverse Colon
TVC	Time-Varying Cox
UNEHS	Unified National Electronic Health System
VIF	Variance Inflation Factor

## References

[1] Sung H., Ferlay J., Siegel R.L., Laversanne M., Soerjomataram I., Jemal A., Bray F. (2021). Global Cancer Statistics 2020: GLOBOCAN Estimates of Incidence and Mortality Worldwide for 36 Cancers in 185 Countries. CA Cancer J. Clin..

[2] Bray F., Ferlay J., Soerjomataram I., Siegel R.L., Torre L.A., Jemal A. (2018). Global Cancer Statistics 2018: GLOBOCAN Estimates of Incidence and Mortality Worldwide for 36 Cancers in 185 Countries. CA Cancer J. Clin..

[3] Mauyenova D., Zhadykova Y., Khozhayev A., Turebayev D., Kulmirzayeva D., Urazova S., Nurtazinova G., Kuandykov Y., Amanshayeva A., Sakhanov S. (2021). Trends of Colorectal Cancer Incidence in Kazakhstan. Asian Pac. J. Cancer Prev..

[4] Mauyenova D., Axarin A., Telmanova Z., Baibusunova A., Bilyalova Z., Igissinova G., Bukeyeva Z., Zhantureyeva A., Kozhakhmetova Z., Kuandykov Y. (2022). Colorectal Cancer Mortality in Kazakhstan: Spatio-Temporal Epidemiological Assessment. Asian Pac. J. Cancer Prev..

[5] Akhmedullin R., Aimyshev T., Zhakhina G., Yerdessov S., Beyembetova A., Ablayeva A., Biniyazova A., Seyil T., Abdukhakimova D., Segizbayeva A. (2024). In-Depth Analysis and Trends of Cancer Mortality in Kazakhstan: A Joinpoint Analysis of Nationwide Healthcare Data 2014–2022. BMC Cancer.

[6] Zhylkaidarova A., Kaidarova D., Batyrbekov K., Shatkovskaya O., Begimbetova D. (2021). Trends of Colorectal Cancer Prevalence in Kazakhstan Related to Screening. Clin. Endosc..

[7] Beyembetova A., Ablayeva A., Akhmedullin R., Abdukhakimova D., Biniyazova A., Gaipov A. (2025). National Electronic Oncology Registry in Kazakhstan: Patient’s Journey. Epidemiol. Health Data Insights.

[8] Gusmanov A., Zhakhina G., Yerdessov S., Sakko Y., Mussina K., Alimbayev A., Syssoyev D., Sarria-Santamera A., Gaipov A. (2023). Review of the Research Databases on Population-Based Registries of Unified Electronic Healthcare System of Kazakhstan (UNEHS): Possibilities and Limitations for Epidemiological Research and Real-World Evidence. Int. J. Med. Inform..

[9] Ludvigsson J.F., Appelros P., Askling J., Byberg L., Carrero J.-J., Ekström A.M., Ekström M., Smedby K.E., Hagström H., James S. (2021). Adaptation of the Charlson Comorbidity Index for Register-Based Research in Sweden. Clin. Epidemiol..

[10] Quan H., Sundararajan V., Halfon P., Fong A., Burnand B., Luthi J.-C., Saunders L.D., Beck C.A., Feasby T.E., Ghali W.A. (2005). Coding Algorithms for Defining Comorbidities in ICD-9-CM and ICD-10 Administrative Data. Med. Care.

[11] Van Der Hulst H.C., Van Der Bol J.M., Bastiaannet E., Portielje J.E.A., Dekker J.W.T. (2024). Age-Specific Impact of Comorbidity on Postoperative Outcomes in Older Patients with Colorectal Cancer. J. Geriatr. Oncol..

[12] Luhn P., Kuk D., Carrigan G., Nussbaum N., Sorg R., Rohrer R., Tucker M.G., Arnieri B., Taylor M.D., Meropol N.J. (2019). Validation of Diagnosis Codes to Identify Side of Colon in an Electronic Health Record Registry. BMC Med. Res. Methodol..

[13] Majek O., Gondos A., Jansen L., Emrich K., Holleczek B., Katalinic A., Nennecke A., Eberle A., Brenner H., The GEKID Cancer Survival Working Group (2013). Sex Differences in Colorectal Cancer Survival: Population-Based Analysis of 164,996 Colorectal Cancer Patients in Germany. PLoS ONE.

[14] Wang C.B., Shahjehan F., Merchea A., Li Z., Bekaii-Saab T.S., Grothey A., Colibaseanu D.T., Kasi P.M. (2019). Impact of Tumor Location and Variables Associated with Overall Survival in Patients with Colorectal Cancer: A Mayo Clinic Colon and Rectal Cancer Registry Study. Front. Oncol..

[15] (2025). Demographic Statistics Bureau of National Statistics of the Agency for Strategic Planning and Reforms of the Republic of Kazakhstan. https://stat.gov.kz/ru/industries/social-statistics/demography/publications/377512/.

[16] Ahmad O.B., Boschi Pinto C., Lopez A.D. (2001). Age Standardization of Rates: A New WHO Standard.

[17] Arnold M., Sierra M.S., Laversanne M., Soerjomataram I., Jemal A., Bray F. (2017). Global Patterns and Trends in Colorectal Cancer Incidence and Mortality. Gut.

[18] Franko J., Shi Q., Meyers J.P., Maughan T.S., Adams R.A., Seymour M.T., Saltz L., Punt C.J.A., Koopman M., Tournigand C. (2016). Prognosis of Patients with Peritoneal Metastatic Colorectal Cancer given Systemic Therapy: An Analysis of Individual Patient Data from Prospective Randomised Trials from the Analysis and Research in Cancers of the Digestive System (ARCAD) Database. Lancet Oncol..

[19] Klaver Y.L.B., Simkens L.H.J., Lemmens V.E.P.P., Koopman M., Teerenstra S., Bleichrodt R.P., De Hingh I.H.J.T., Punt C.J.A. (2012). Outcomes of Colorectal Cancer Patients with Peritoneal Carcinomatosis Treated with Chemotherapy with and without Targeted Therapy. Eur. J. Surg. Oncol. (EJSO).

[20] Furukawa S., Hiraki M., Kimura N., Okuyama K., Kohya N., Sakai M., Kawaguchi A., Ikubo A., Samejima R. (2025). The Clinical Impact of Intraoperative Bleeding on Peritoneal Recurrence after Surgery for Stage II to III Colorectal Cancer. Asian J. Surg..

[21] Bhattacharya S. (2022). Colorectal Cancer Diagnosis and Therapeutic Updates.

[22] Emile S.H., Horesh N., Strassmann V., Garoufalia Z., Gefen R., Zhou P., Wexner S.D. (2025). A National Cancer Database Analysis of the Characteristics and Outcome of Colon Cancer According to Type of Preexisting Adenoma. Clin. Color. Cancer.

[23] Wu X., Lin H., Li S. (2019). Prognoses of Different Pathological Subtypes of Colorectal Cancer at Different Stages: A Population-Based Retrospective Cohort Study. BMC Gastroenterol..

[24] Morishima T., Matsumoto Y., Koeda N., Shimada H., Maruhama T., Matsuki D., Nakata K., Ito Y., Tabuchi T., Miyashiro I. (2019). Impact of Comorbidities on Survival in Gastric, Colorectal, and Lung Cancer Patients. J. Epidemiol..

[25] Michalopoulou E., Matthes K.L., Karavasiloglou N., Wanner M., Limam M., Korol D., Held L., Rohrmann S. (2021). Impact of Comorbidities at Diagnosis on the 10-Year Colorectal Cancer Net Survival: A Population-Based Study. Cancer Epidemiol..

[26] Rubio F.J., Alvares D., Redondo-Sanchez D., Marcos-Gragera R., Sánchez M.-J., Luque-Fernandez M.A. (2022). Bayesian Variable Selection and Survival Modeling: Assessing the Most Important Comorbidities That Impact Lung and Colorectal Cancer Survival in Spain. BMC Med. Res. Methodol..

[27] Tamraz M., Al Ghossaini N., Temraz S. (2024). Optimization of Colorectal Cancer Screening Strategies: New Insights. World J. Gastroenterol..

[28] Suenghataiphorn T., Kulthamrongsri N., Danpanichkul P., Saowapa S., Polpichai N., Thongpiya J. (2024). Impact of Dementia in Colorectal Cancer Patients: United States Population-Based Cohort Study. Korean J. Gastroenterol..

[29] Cho H.J., Lee H.S., Kang J. (2024). Synergistic Prognostic Impact of Hemoglobin and Skeletal Muscle Index in Patients with Colorectal Cancer. Clin. Nutr. ESPEN.

[30] Balboa-Barreiro V., Pértega-Díaz S., García-Rodríguez T., González-Martín C., Pardeiro-Pértega R., Yáñez-González-Dopeso L., Seoane-Pillado T. (2024). Colorectal Cancer Recurrence and Its Impact on Survival after Curative Surgery: An Analysis Based on Multistate Models. Dig. Liver Dis..

[31] Kang J.H., Son I.T., Yoon S.N., Ihm J.S., Kang B.M., Kim J.W. (2024). Impact of COVID-19 Pandemic on the Clinical and Pathologic Characteristics of Colorectal Cancer: A Retrospective Multicenter Study in South Korea. Cancer Manag. Res..

[32] Loroña N.C., Himbert C., Ose J., Cohen S.A., Strehli I., Ulrich C.M., Cobos S., Jean-Baptiste E., Bloomer A.M., Figueiredo J.C. (2025). Alcohol Consumption and Smoking History at the Time of Diagnosis and the Risk of Colorectal Cancer Recurrence and Mortality: Results from the ColoCare Study. Cancer Epidemiol. Biomark. Prev..

[33] Sada H., Hinoi T., Niitsu H., Ohdan H., Yamamoto S., Endo S., Hida K., Kinugasa Y., Enomoto T., Maruyama S. (2024). Right-Sided Versus Left-Sided Colorectal Cancer in Elderly Patients: A Sub-Analysis of a Large Multicenter Case–Control Study in Japan. Surg. Today.

[34] Osterman E., Syriopoulou E., Martling A., Andersson T.M.-L., Nordenvall C. (2024). Despite Multi-Disciplinary Team Discussions the Socioeconomic Disparities Persist in the Oncological Treatment of Non-Metastasized Colorectal Cancer. Eur. J. Cancer.

[35] Katayama E.S., Woldesenbet S., Tsilimigras D., Munir M.M., Endo Y., Huang E., Cunningham L., Harzman A., Gasior A., Husain S. (2024). Inflammatory Bowel Disease-Associated Colorectal Cancer Negatively Affects Surgery Outcomes and Health Care Costs. Surgery.

[36] Asghari-Jafarabadi M., Wilkins S., Plazzer J.P., Yap R., McMurrick P.J. (2024). Prognostic Factors and Survival Disparities in Right-Sided Versus Left-Sided Colon Cancer. Sci. Rep..

[37] Schmocker R.K., Enomoto L.M., Low G.K., McLoughlin J.M., Casillas M.A., Antill A.C., Heidel R.E., Russ A.J. (2024). Further Distance from Treating Facility Is Associated with Advanced Colon Cancer at Presentation and Increased Mortality. Am. Surg..

[38] Alese O.B., Zhou W., Jiang R., Zakka K., Huang Z., Okoli C., Shaib W.L., Akce M., Diab M., Wu C. (2021). Predictive and Prognostic Effects of Primary Tumor Size on Colorectal Cancer Survival. Front. Oncol..

[39] Al Khashali H., Ray R., Darweesh B., Wozniak C., Haddad B., Goel S., Seidu I., Khalil J., Lopo B., Murshed N. (2024). Amyloid Beta Leads to Decreased Acetylcholine Levels and Non-Small Cell Lung Cancer Cell Survival via a Mechanism That Involves P38 Mitogen-Activated Protein Kinase and Protein Kinase C in a P53-Dependent and -Independent Manner. Int. J. Mol. Sci..

[40] Kolobova E., Petrushanko I., Mitkevich V., Makarov A.A., Grigorova I.L. (2024). β-Amyloids and Immune Responses Associated with Alzheimer’s Disease. Cells.

[41] Shi J., Hubbard A.E., Fong N., Pirracchio R. (2024). Implicit Bias in Critical Care Data: Factors Affecting Sampling Frequencies and Missingness Patterns of Clinical and Biological Variables in ICU Patients. medRXiv.

[42] Beaulieu-Jones B.K., Lavage D.R., Snyder J.W., Moore J.H., Pendergrass S.A., Bauer C.R. (2018). Characterizing and Managing Missing Structured Data in Electronic Health Records: Data Analysis. JMIR Med. Inf..

[43] Beaulieu-Jones B.K., Moore J.H. (2017). The Pooled Resource Open-Access Als Clinical Trials Consortium Missing Data Imputation in the Electronic Health Record Using Deeply Learned Autoencoders. Proceedings of the Biocomputing 2017.

[44] Botsis T., Hartvigsen G., Chen F., Weng C. (2010). Secondary Use of EHR: Data Quality Issues and Informatics Opportunities. Summit Transl. Bioinform..

[45] Kharrazi H., Wang C., Scharfstein D. (2014). Prospective EHR-Based Clinical Trials: The Challenge of Missing Data. J. Gen. Intern. Med..

[46] Wells B.J., Nowacki A.S., Chagin K., Kattan M.W. (2013). Strategies for Handling Missing Data in Electronic Health Record Derived Data. eGEMs.

[47] Sharafoddini A., Dubin J.A., Maslove D.M., Lee J. (2019). A New Insight Into Missing Data in Intensive Care Unit Patient Profiles: Observational Study. JMIR Med. Inf..

[48] Seaman S.R., White I.R. (2013). Review of Inverse Probability Weighting for Dealing with Missing Data. Stat. Methods Med. Res..

