# Supplementary material for: Epidemiological Insights into Colorectal Cancer Survival in Kazakhstan (2014–2023): A Retrospective Analysis Using the National Electronic Registry of Oncological Patients
Source: Cancers (Basel). 2025 Jul 14;17(14):2336. doi: 10.3390/cancers17142336 (PMC12293920; doi:10.3390/cancers17142336)
Supplement: Supplementary file 1 [file cancers-17-02336-s001.zip › cancers-3706552-supplementary.pdf]

Supplementary Materials

Table S1. *The ICD-10 codes used to identify colorectal cancer patients.*

**Condition**	**ICD-10 Codes and explanations**
**Colorectal cancer**	C18 – Malignant neoplasm of colon
	C18.0 – Malignant neoplasm of cecum
	C18.1 – Malignant neoplasm of appendix
	C18.2 – Malignant neoplasm of ascending colon
	C18.3 – Malignant neoplasm of hepatic flexure
	C18.4 – Malignant neoplasm of transverse colon
	C18.5 – Malignant neoplasm of splenic flexure
	C18.6 – Malignant neoplasm of descending colon
	C18.7 – Malignant neoplasm of sigmoid colon
	C18.8 – Malignant neoplasm of overlapping sites of colon
	C18.9 – Malignant neoplasm of colon, unspecified
	C19 – Malignant neoplasm of rectosigmoid junction
	C20 – Malignant neoplasm of rectum
	C21 – Malignant neoplasm of anus and anal canal
	C21.0 – Malignant neoplasm of anus, unspecified
	C21.1 – Malignant neoplasm of anal canal
	C21.2 – Malignant neoplasm of cloacogenic zone
	C21.8 – Malignant neoplasm of overlapping sites of rectum, anus, and anal canal
	C21.9 – Malignant neoplasm of anus, unspecified
	C78.5 - Secondary malignant neoplasm of large intestine and rectum
	D01 – Carcinoma in situ of colon, rectum, and anus
	D01.0 – Carcinoma in situ of colon
	D01.1 – Carcinoma in situ of rectosigmoid junction
	D01.2 – Carcinoma in situ of rectum
	D01.3 – Carcinoma in situ of anus and anal canal
	D01.40 - Carcinoma in situ of unspecified part of intestine

	D01.49 - Carcinoma in situ of other parts of intestine
	D12 – Benign neoplasm of colon, rectum, anus, and anal canal
	D12.0 – Benign neoplasm of cecum
	D12.1 – Benign neoplasm of appendix
	D12.2 – Benign neoplasm of ascending colon
	D12.3 – Benign neoplasm of transverse colon
	D12.4 – Benign neoplasm of descending colon
	D12.5 – Benign neoplasm of sigmoid colon
	D12.6 – Benign neoplasm of colon, unspecified
	D12.7 – Benign neoplasm of rectosigmoid junction
	D12.8 – Benign neoplasm of rectum
	D12.9 – Benign neoplasm of anus and anal canal
	D37 – Neoplasm of uncertain or unknown behavior of digestive organs
	D37.3 – Neoplasm of uncertain behavior of colon
	D37.4 – Neoplasm of uncertain behavior of rectum
	D37.5 – Neoplasm of uncertain behavior of anus and anal canal

Table S2. *ICD-10 codes used to define comorbidities*.

Commorbid condition	ICD-10 code	Weighted score in CCI
**Myocardial infarction**	I21, I22, I25.2	1
**Congestive heart failure**	I11.0, I13.0, I13.2, I25.5, I42.0, I42.6, I42.7, I42.8, I42.9, I43, I50	1
**Peripheral vascular disease**	I70, I71, I73.1, I73.8, I73.9, I77.1, I79.0, I79.2, K55,	1
**Cerebrovascular disease**	G45, I60, I61, I62, I63, I64, I67, I69	1
**Chronic obstructive pulmonary disease (COPD)**	J43, J44	1

**Other chronic pulmonary disease**	J41, J42, J45, J46, J47, J60, J61, J62, J63, J64, J65, J66, J67, J68, J69, J70	1
**Rheumatic disease**	M05; M06, M12.3, M07.0–3, M08, M13, M30, M31.3-M31.6, M32, M33, M34, M35.0, M35.1, M35.3, M45-46	1
**Dementia**	F00, F01, F02, F03, F05.1, G30, G31.1, G31.9	1
**Hemiplegia, tetraplegia**	G11.4, G80, G81, G82, G83.0-G83.3, G83.8	2
**Diabetes**	E10.0, E10.1, and other forms of diabetes without end-stage organ damage E11.0-E11.1, E12.0-E12.1, E13.0-E13.1, E14.0-E14.1	1
**Diabetes with end organ damage**	E10.2, E10.3, E10.4, E10.5, E10.7, and other forms of diabetes with same End-stage organ damage E11.2-E11.7, E12.2-E12.7, E13.2-E13.7, E14.2-E14.7	2
**Moderate or severe kidney disease**	N03.2-N03.7, N05.2-N05.7, N11, N18, N19, N25.0, I12.0, I13.1, Q61.1-Q61.4, Z49, Z94.0, Z99.2	2
**Mild liver disease**	B15-B19, K70.9, K73, K74.6, K70.3, K75.4	1
**Moderate or severe liver disease**	R18 (+Any code for mild liver disease in combination with ascites)<br><br>I85.0, I85.9, I98.2, I98.3	3
**(Peptic) Ulcer disease**	K25, K26, K27 , K28	1
**Any malignancy, including leukemia and lymphoma***	C00-C97, and not (C18, C19, C21, C87)	2
**HIV/AIDS**	B20, B21, B22, B23, B24, F02.4, O98.7, R75, Z11.4, Z21.9, Z71.1	6
**Inflammatory Bowel Disease**	K50.0, K50.1, K50.8, K50.9, K51.0, K51.2, K51.3, K51.5, K51.8, K51.9, K52.8	not applicable

Table S3. *The number of colorectal cancer patients with different histological subtypes.*

**Histological subtype**	**ICD-O-3 code**	***n***
**Classical adenocarcinoma**	8140/2 Adenocarcinoma in situ, NOS	49
	8140/3: Adenocarcinoma, NOS	23,574
	8140/6 Adenocarcinoma, metastatic, NOS	93
	8141/3: Scirrhous adenocarcinoma	16
	8143/3: Superficial spreading adenocarcinoma	11
	8144/3: Adenocarcinoma, intestinal type	1684
	8210/2 Adenocarcinoma in situ in adenomatous polyp	33
	8210/3: Adenocarcinoma in adenomatous polyp	176
	8211/3: Tubular adenocarcinoma	24

	8220/3: Adenocarcinoma in adenomatous polyposis coli	79
	8221/3: Adenocarcinoma in multiple adenomatous polyps	3
	8260/3: Papillary adenocarcinoma, NOS	34
	8261/2 Adenocarcinoma in situ in villous adenoma	1
	8261/3: Adenocarcinoma in villous adenoma	34
	8262/3: Villous adenocarcinoma	4
	8263/2: Adenocarcinoma in situ in tubulovillous adenoma	3
	8263/3: Adenocarcinoma in tubulovillous adenoma	156
	8310/3: Clear cell adenocarcinoma, NOS	6
	8330/3: Follicular adenocarcinoma, NOS	1
	8331/3: Follicular adenocarcinoma well diff	5
	8401/3: Apocrine adenocarcinoma	1
	8410/3: Sebaceous adenocarcinoma	1
	8420/3: Ceruminous adenocarcinoma	1
	8503/3: Intraductal papillary adenocarcinoma with invasion	1
	8525/3: Polymorphous low grade adenocarcinoma	5
	8570/3: Adenocarcinoma with squamous metaplasia	1
**Mucinous adenocarcinoma**	8480/3: Mucinous adenocarcinoma	401
	8481/3: Mucin-producing adenocarcinoma	114
**Signet-ring cell carcinoma**	8490/3: Signet-ring cell carcinoma	87
	8490/6: Metastatic signet ring cell carcinoma	4
**Squamous cell carcinoma**	8070/2: Squamous cell carcinoma in situ, NOS	4
	8070/3: Squamous cell carcinoma, NOS	493
	8070/6: Squamous cell carcinoma, metastatic, NOS	4
	8071/3: Sq. cell carcinoma, keratinizing, NOS	107
	8072/3: Sq. cell carcinoma, lg. cell, non-ker.	115
	8073/3: Sq. cell carcinoma, sm. cell, non-ker.	17
	8074/3: Sq. cell carcinoma, spindle cell	1
	8075/3: Squamous cell carcinoma, adenoid	34
	8076/3: Sq. cell carcinoma, micro-invasive	2
	8077/2: Squamous intraepithelial neoplasia, grade III	3
	8078/3: Squamous cell carcinoma with horn formation	2
	8083/3: Basaloid squamous cell carcinoma	2
**Neoplasms, NOS**	8000/0: Neoplasm, benign	6
	8000/1: Neoplasm, uncertain whether benign or malignant	11
	8000/3: Neoplasm, malignant	1483
	8000/6: Neoplasm, metastatic	6
	8000/9: Neoplasm, malignant, uncertain whether primary or metastatic	3
	8001/0: Tumor cells, benign	2
	8001/1: Tumor cells, uncertain whether benign or malignant	1
	8001/3 :Tumor cells, malignant	507

	8002/3: Malignant tumor, small cell type	2
	8003/3: Malignant tumor, giant cell type	1
**Epithelial neoplasms, NOS**	8010/2: Carcinoma in situ, NOS	12
	8010/3: Carcinoma, NOS	1549
	8010/6: Carcinoma, metastatic, NOS	38
	8010/9: Carcinomatosis	11
	8011/3: Epithelioma, malignant	1
	8012/3: Large cell carcinoma, NOS	2
	8013/3: Large cell neuroendocrine carcinoma	2
	8014/3: Large cell carcinoma with rhabdoid phenotype	1
	8020/3: Carcinoma, undifferentiated type, NOS	168
	8021/3: Carcinoma, anaplastic type, NOS	12
	8030/3: Giant cell and spindle cell carcinoma	2
	8040/1: Tumorlet, NOS	2
	8041/3: Small cell carcinoma, NOS	39
	8043/3: Small cell carcinoma, fusiform cell	1
**Other specified types**	8050/3, 8090/3, 8097/3, 8098/3, 8110/3, 8120/2, 8120/3, 8123/3, 8124/3, 8130/2, 8130/3, 8140/0, 8140/1, 8142/3, 8145/3, 8153/3, 8161/3, 8170/3, 8190/3, 8200/3, 8210/0 , 8211/0, 8220/0 , 8230/3, 8231/3, 8240/1, 8240/3, 8241/3, 8243/3, 8244/3, 8245/3, 8246/3, 8260/0, 8261/1, 8263/0, 8312/3, 8323/3 , 8330/0, 8340/3, 8350/3, 8370/3, 8380/3, 8440/3, 8441/3, 8450/3, 8470/3, 8480/0, 8480/6, 8500/3, 8504/3, 8510/3, 8521/3, 8550/3, 8560/3, 8620/3, 8640/3, 8720/3, 8730/3, 8800/3, 8801/3, 8802/3, 8810/3, 8814/3, 8821/1 , 8850/3, 8851/3, 8852/3, 8890/3, 8891/3, 8930/3, 8933/3, 8940/3, 8960/3, 8963/3, 8980/3, 9070/3, 9120/3 , 9140/3, 9150/3, 9420/3, 9451/3, 9590/3, 9591/3, 9673/3, 9690/3, 9694/3, 9699/3	1210
**Overall**		32,458

Table S4. *ICD-10 codes used to define primary tumor location.*

	Primary tumor location	ICD-10 codes
**1**	**Right colon (ascending colon)**	
	Cecum	C18.0
	Ascending colon	C18.2
	Hepatic flexure	C18.3
**2**	**Transverse colon**	
	Transverse colon	C18.4
**3**	**Left colon (descending colon)**	
	Splenic flexure	C18.5
	Descending colon	C18.6

	Sigmoid colon	C18.7
4	**Rectosigmoid junction**	
	Rectosigmoid junction	C19
5	**Unspecified colon site**	
	Malignant neoplasm of colon	C18
	Malignant neoplasm of appendix	C18.1
	Malignant neoplasm of overlapping sites of colon	C18.8
	Colon unspecified	C18.9
6	**Rectum**	
	Rectum	C20
	Malignant neoplasm of anus and anal canal	C21
	Malignant neoplasm of anus, unspecified	C21.0
	Malignant neoplasm of anal canal	C21.1
	Malignant neoplasm of cloacogenic zone	C21.2
	Malignant neoplasm of overlapping sites of rectum, anus and anal canal	C21.8
7	**Not applicable**	
	Benign, in situ, uncertain behavior neoplasms	D01, D01.0, D01.1, D01.2, D01.3, D01.4, D01.5, D01.7, D01.9, D12, D12.0, D12.1, D12.2, D12.3, D12.4, D12.5, D12.6, D12.7, D12.8, D12.9, D37, D37.0, D37.1, D37.2, D37.3, D37.4, D37.5, D37.6, D37.7, D37.9

Table S5. *Association between demographic and medical parameters and all-cause mortality rates in colorectal cancer patients (2014–2023).*

	**First year of follow-up**		**Subsequent years**	
	**Hazard Ratio (95% CI)**	***p*-Value**	**Hazard Ratio (95% CI)**	***p*-Value**
Age group				
18-44	reference		reference	
45-54	0.92 (0.85-0.99)†	**0.031**†	1.03 (0.91-1.17)	0.606
55-64	0.89 (0.83-0.95)†	**0.001**†	1.12 (1.00-1.26)	**0.034**
65-74	0.88 (0.82-0.94)†	**<0.001**†	1.33 (1.19-1.48)	**<0.001**
≥75	0.86 (0.80-0.92)†	**<0.001**†	2.01 (1.80-2.26)	**<0.001**
Sex				
Female	reference		reference	
Male	1.14 (1.10-1.19)	**<0.001**	1.07 (0.98-1.17)†	0.116†
Ethnicity				
Kazakh	reference		reference	
Other	1.00 (0.97-1.03)§	0.942§	0.98 (0.92-1.04)	0.511
Russian	0.91 (0.89-0.93) †	**<0.001**†	1.03 (0.98-1.09)	0.283
Primary tumor location				

Left colon (descending colon)	reference		reference	
Rectosigmoid junction	1.09 (1.05-1.13)†	**<0.001**†	0.88 (0.76-1.01)§	0.084§
Rectum	1.21 (1.17-1.25)†	**<0.001**†	0.87 (0.78-0.97)†	**0.014**†
Right colon (ascending colon)	1.07 (1.04-1.11)†	**<0.001**†	0.85 (0.73-0.98)§	**0.032**§
Transverse colon	1.03 (0.98-1.09)§	0.248§	0.86 (0.64-1.15)†	0.300†
Unspecified colon site	0.94 (0.90-0.99)§	**0.022**§	1.10 (0.85-1.41)§	0.466§
Histological subtypes				
Classical adenocarcinoma	reference		reference	
Epithelial neoplasms, NOS	0.89 (0.85-0.92)†	**<0.001**†	0.85 (0.67-1.07)§	0.167§
Mucinous adenocarcinoma	1.21 (1.07-1.37)†	**0.002**†	1.00 (0.72-1.39)§	0.977§
Neoplasms, NOS	0.86 (0.83-0.89)†	**<0.001**†	0.81 (0.69-0.96)†	**0.017**†
Other specified types	0.96 (0.92-1.01)†	0.101†	0.75 (0.55-1.04)§	0.083§
Signet-ring cell carcinoma	1.04 (0.88-1.22)§	0.646§	0.48 (0.16-1.46)§	0.196§
Squamous cell carcinoma, NOS	1.09 (0.98-1.20)§	0.100§	0.91 (0.69-1.20)§	0.515§
CCI group				
No comorbidity (0)	reference		reference	
Low (1–2)	0.81 (0.77-0.85)	**<0.001**	1.48 (1.34-1.63)†	**<0.001**†
Moderate (3–4)	0.72 (0.66-0.79)	**<0.001**	1.78 (1.51-2.11)†	**<0.001**†
High (≥5)	0.65 (0.53-0.79)	**<0.001**	2.04 (1.48-2.82)†	**<0.001**†

Note: Adjusted hazard ratios (aHRs) and 95% confidence intervals (CIs) are derived from multivariable Cox regression models, adjusted for all covariates listed. Models were stratified by stage due to proportional hazards (PH) violation. Time-varying Cox (TVC) models were used where applicable. Results are presented separately for the first year of follow-up and the subsequent years to account for time-dependent effects. † PH assumption violated; TVC estimate reported. § PH assumption not violated, but TVC applied for consistency across variable categories. **Bold *p*-values** indicate statistical significance ($p < 0.05$).

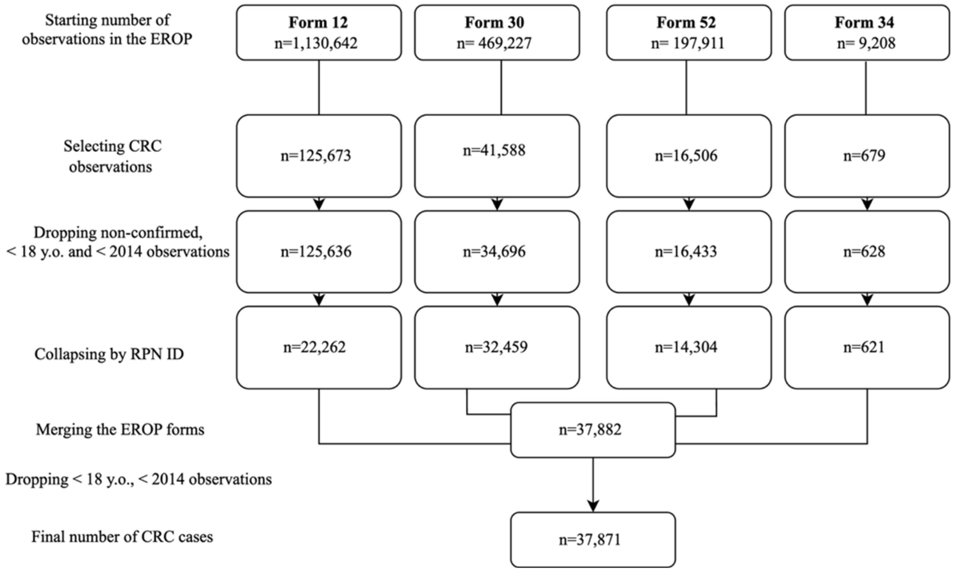

Figure S1. Colorectal Cancer Cohort Selection Diagram.

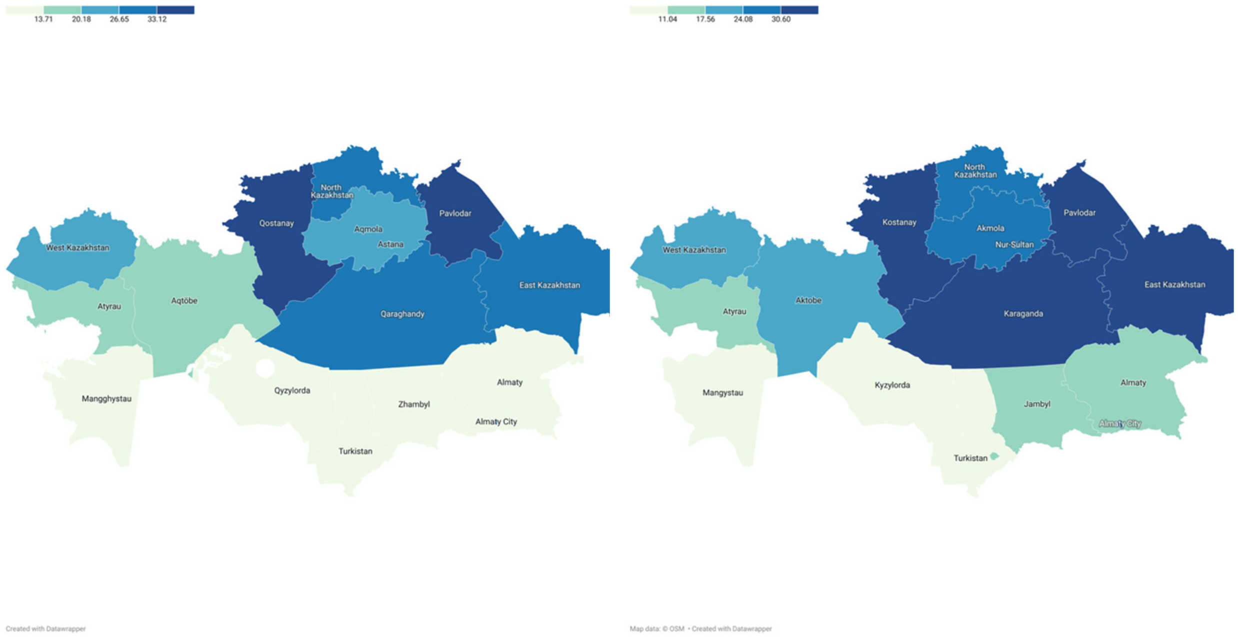

(**a**) (**b**)

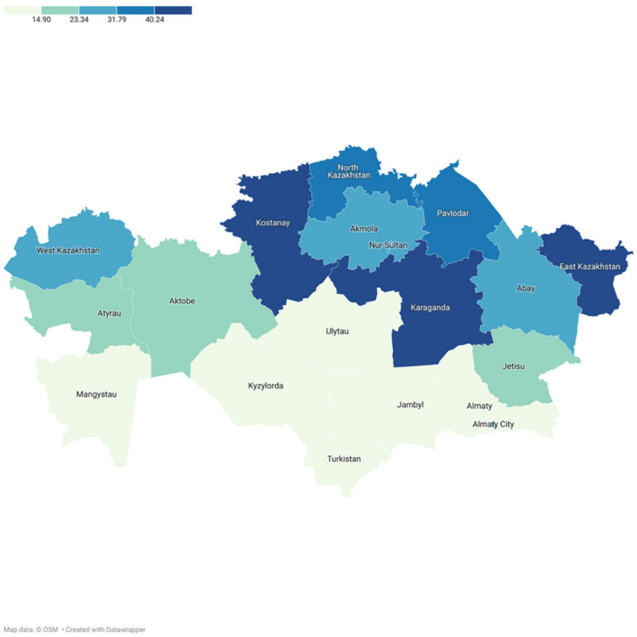

(c)

Figure S2. Average Incidence Rate of colorectal cancer in the regions of Kazakhstan per 100, 000 population: (**a**) 2014-2017, (**b**) 2018-2021, (**c**) 2022-2023.

Table S6. *Incidence rate of colorectal cancer in the regions of Kazakhstan by years in 2014-2023.*

Region\years	2014	2015	2016	2017	2018	2019	2020	2021	2022	2023
Abay region									26.02	28.08
Akmola region	21.37	23.54	30.89	25.02	23.46	31.24	27.35	22.91	25.16	27.92
Aktobe region	14.97	11.96	18.10	24.44	18.42	19.43	15.66	17.89	14.20	18.42
Almaty region	11.45	17.03	13.76	11.66	13.08	12.67	9.69	14.12	14.01	11.66
Almaty city	22.45	26.60	24.14	24.87	26.41	25.66	26.80	27.62	24.82	23.87
Astana city	17.03	17.72	30.36	23.31	28.00	25.32	20.92	22.98	27.02	30.17
Atyrau region	12.18	12.93	17.14	13.35	14.03	15.32	14.43	16.15	16.30	18.04
East Kazakhstan region	28.55	29.75	35.85	18.11	42.31	29.20	29.14	32.30	48.11	49.27
Jambyl region	10.55	9.43	12.68	11.03	9.55	11.45	16.06	14.52	11.62	11.72
Jetisu region									15.89	16.75
Karaganda region	22.21	24.12	33.55	30.05	27.85	29.34	27.11	43.39	54.01	38.50
Kostanay region	29.64	33.33	36.10	59.32	50.26	33.91	31.07	33.23	38.97	43.56
Kyzylorda region	7.51	6.46	9.37	12.61	7.99	8.39	8.66	10.36	9.29	10.62
Mangystau region	9.87	7.45	12.75	11.65	10.30	10.74	8.59	8.21	10.71	15.44
North Kazakhstan region	31.75	32.10	33.41	29.63	28.42	32.12	32.08	27.42	34.85	34.39
Pavlodar region	33.29	30.13	36.70	38.25	31.96	37.33	29.28	33.66	34.54	42.28
Shymkent city					18.97	11.82	13.07	11.34	9.34	9.86
South Kazakhstan region	5.80	7.79	7.17	8.17						
Turkistan region					1.82	4.91	5.57	5.78	6.37	6.52
Ulutay region									1.81	13.09
West Kazakhstan region	19.46	21.63	29.11	22.67	22.17	25.21	18.21	21.86	25.67	21.86

Note: Color scale represents regional variation in colorectal cancer incidence across Kazakhstan from 2014 to 2023, based on administrative divisions. Blue indicates lower incidence, while red indicates higher incidence (per 100,000 population).

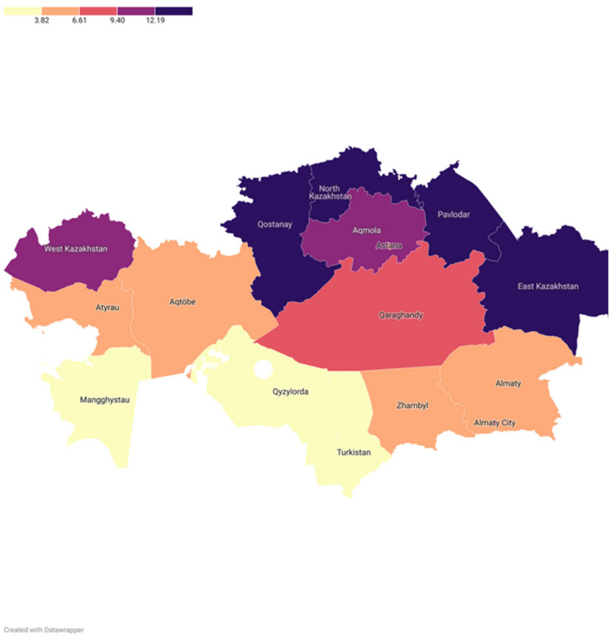

(**a**)

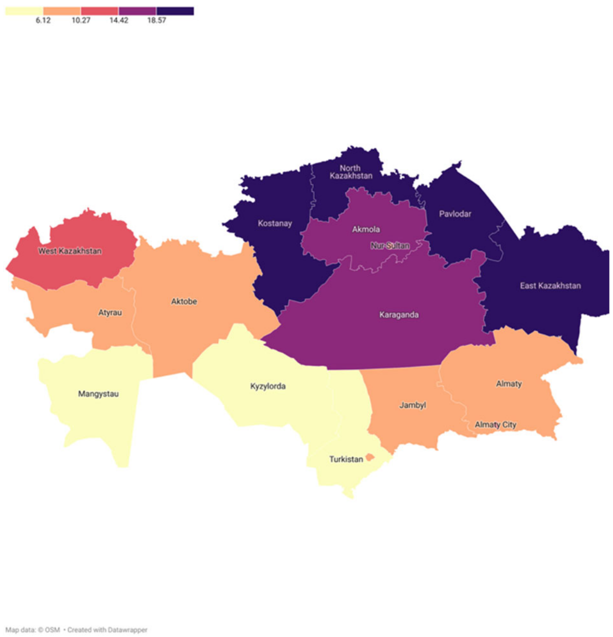

(**b**)

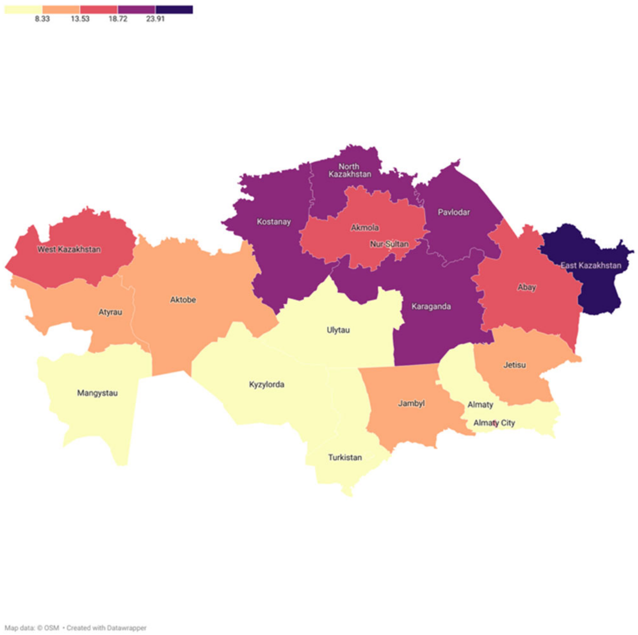

(c)

Figure S3. Average All-Cause Mortality Rate of colorectal cancer in the regions of Kazakhstan per 100, 000 population: (**a**) 2014-2017, (**b**) 2018-2021, (**c**) 2022-2023.

Table S7. *All-cause mortality rate of colorectal cancer cohort in the regions of Kazakhstan by years in 2014-2023.*

Region\years	2014	2015	2016	2017	2018	2019	2020	2021	2022	2023
Abay region									16.37	18.56
Akmola region	4.08	11.09	13.96	14.14	13.70	14.40	15.92	15.13	15.50	17.39
Aktobe region	0.86	3.02	7.27	7.17	9.85	8.80	9.13	13.12	10.08	10.92
Almaty region	2.68	4.50	6.32	6.61	6.86	7.73	7.90	7.27	7.51	7.31
Almaty city	4.43	7.81	10.68	10.44	11.02	11.27	13.32	16.55	13.04	15.17
Astana city	2.02	3.68	9.90	12.60	11.88	10.42	11.83	14.03	12.83	13.29
Atyrau region	2.96	4.93	7.99	6.35	6.22	6.88	8.75	9.51	8.73	10.88
East Kazakhstan region	6.53	13.77	12.57	17.25	17.97	17.04	18.23	21.56	28.70	29.51
Jambyl region	3.03	2.72	5.58	4.48	5.09	6.13	6.97	9.36	8.07	8.77
Jetisu region									8.73	8.45
Karaganda region	2.77	6.59	11.06	10.79	12.11	12.71	16.57	20.68	23.61	21.50
Kostanay region	4.20	10.09	16.35	19.16	19.92	22.30	22.98	25.68	22.06	24.79
Kyzylorda region	0.00	0.40	1.56	2.19	1.78	3.26	3.34	6.82	5.91	7.16
Mangystau region	0.50	1.30	3.15	4.14	4.48	4.93	4.93	5.75	6.61	6.44
North Kazakhstan region	6.45	11.75	15.38	18.74	14.03	22.32	20.89	18.16	20.13	20.30
Pavlodar region	6.50	14.53	17.03	21.84	22.28	21.26	23.16	22.57	23.42	23.46
Shymkent city					11.53	8.70	11.27	7.50	7.05	7.12
South Kazakhstan region	1.20	3.27	3.60	4.62						
Turkistan region					0.96	1.35	2.71	2.87	3.18	3.10
Ulutay region									1.81	4.97
West Kazakhstan region	4.95	8.68	11.89	13.66	14.17	11.92	15.02	14.47	14.73	14.33

Note: Color scale represents regional variation in colorectal cancer mortality across Kazakhstan from 2014 to 2023, based on administrative divisions. Blue indicates lower mortality, while red indicates higher mortality (per 100,000 population).

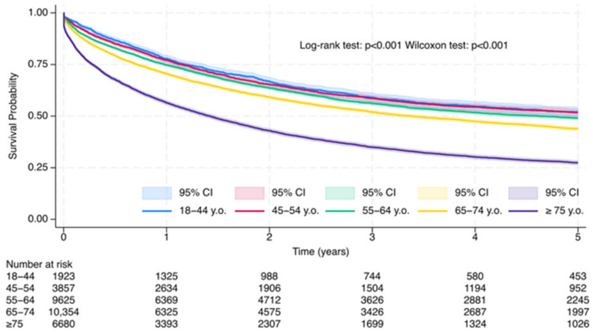

(**a**)

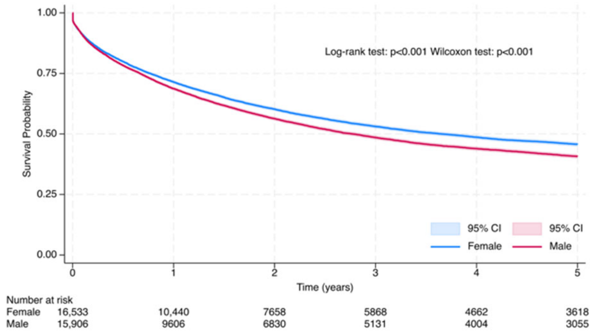

(**b**)

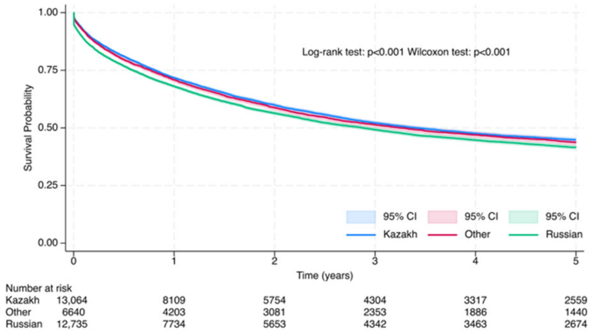

(**c**)

Figure S4. Kaplan-Meier survival curves due to all-cause mortality in colorectal cancer patients based on demographic characteristics: age group (**a**); sex (**b**) and ethnicity (**c**) during a 5-year follow-up.

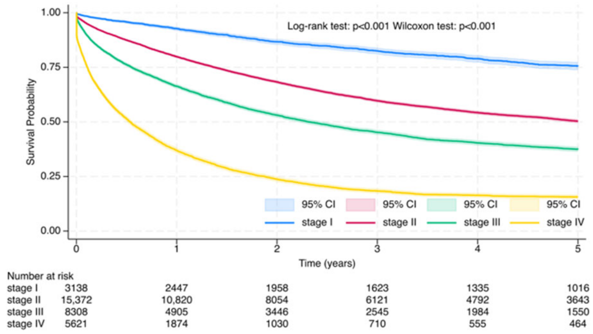

(**a**)

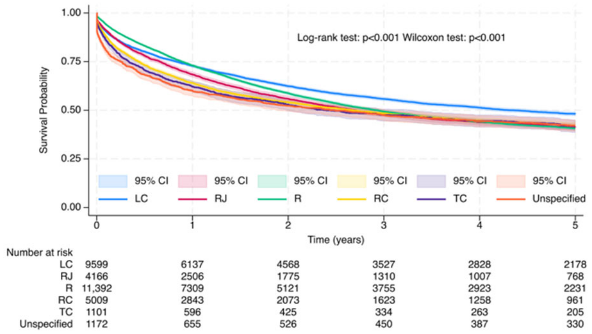

(**b**)

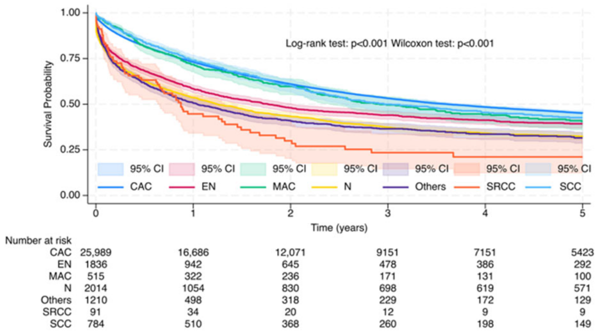

(**c**)

Figure S5. Kaplan-Meier survival curves due to all-cause mortality in colorectal cancer patients based on medical characteristics: stage (**a**); primary tumor location (**b**) and histological subtype (**c**) during 5-year follow-up.

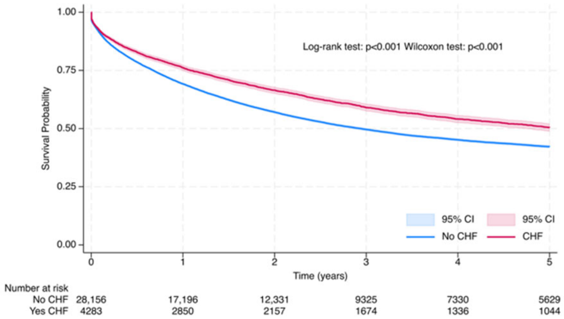

(**a**)

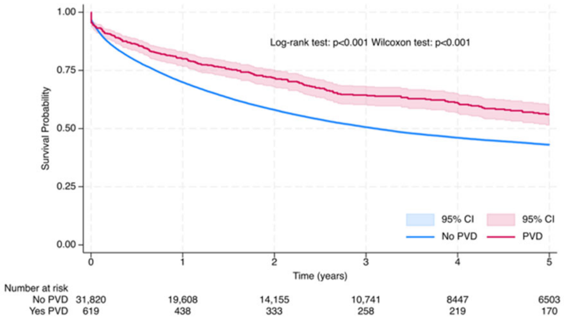

(**b**)

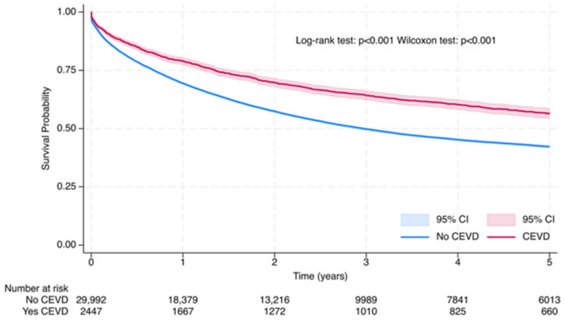

(**c**)

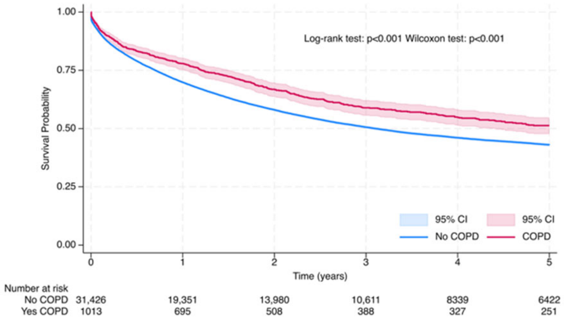

(**d**)

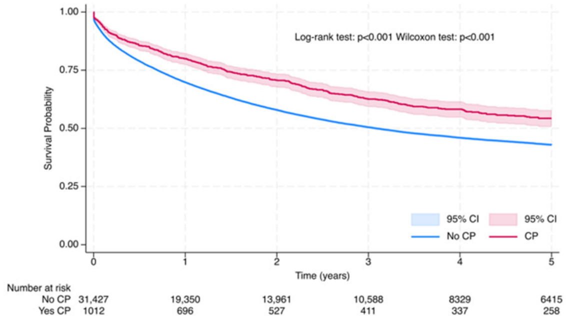

(**e**)

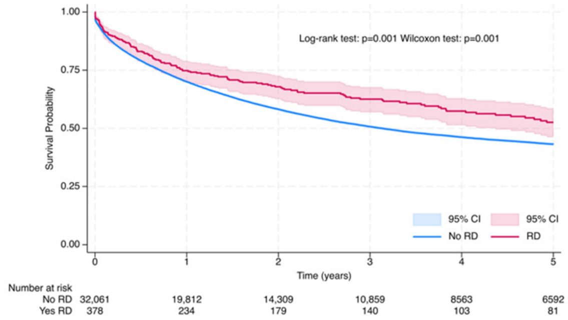

(**f**)

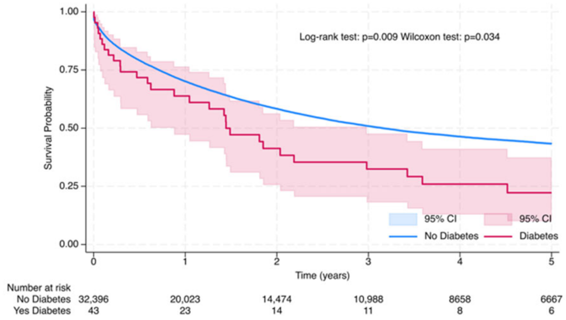

(**g**)

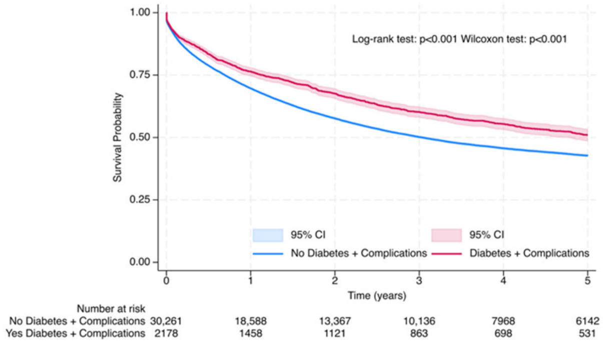

(**h**)

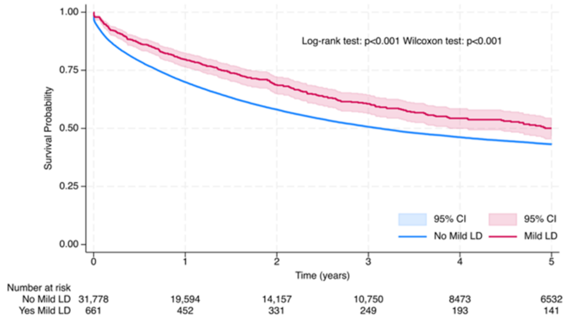

(**i**)

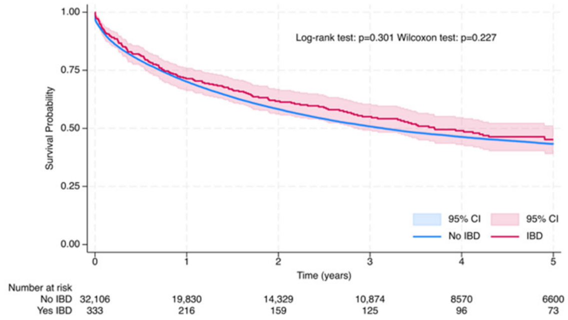

(**j**)

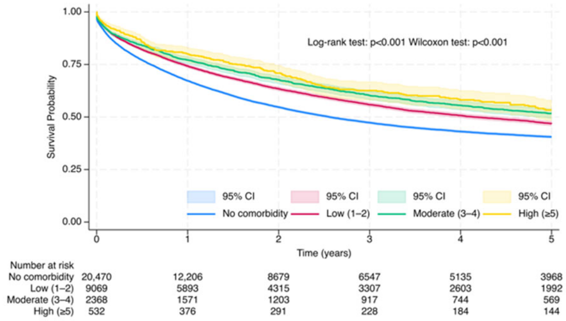

(**k**)

Figure S6. Kaplan-Meier survival curves due to all-cause mortality in colorectal cancer patients based on comorbidities and CCI group: congestive heart failure (**a**);

peripheral vascular disease (**b**); cerebrovascular disease (**c**); chronic obstructive pulmonary disease (**d**); other chronic pulmonary disease (**e**); rheumatic disease (f); diabetes (**g**); diabetes with end organ damage (**h**); mild liver disease (**i**); inflammatory bowel disease (**j**); CCI group (**k**) during 5-year follow up.
